# Chemoreceptor proteins in the Caribbean spiny lobster, *Panulirus argus*: Expression of Ionotropic Receptors, Gustatory Receptors, and TRP channels in two chemosensory organs and brain

**DOI:** 10.1371/journal.pone.0203935

**Published:** 2018-09-21

**Authors:** Mihika T. Kozma, Manfred Schmidt, Hanh Ngo-Vu, Shea D. Sparks, Adriano Senatore, Charles D. Derby

**Affiliations:** 1 Neuroscience Institute, Georgia State University, Atlanta, Georgia, United States of America; 2 Department of Biology, University of Toronto Mississauga, Ontario, Canada; INRA-UPMC, FRANCE

## Abstract

The spiny lobster, *Panulirus argus*, has two classes of chemosensilla representing “olfaction” and “distributed chemoreception,” as is typical for decapod crustaceans. Olfactory sensilla are found exclusively on antennular lateral flagella and are innervated only by olfactory receptor neurons (ORNs) that project into olfactory lobes organized into glomeruli in the brain. Distributed chemoreceptor sensilla are found on all body surfaces including the antennular lateral flagella (LF) and walking leg dactyls (dactyls), and are innervated by both chemoreceptor neurons (CRNs) and mechanoreceptor neurons that project into somatotopically organized neuropils. Here, we examined expression of three classes of chemosensory genes in transcriptomes of the LF (with ORNs and CRNs), dactyls (with only CRNs), and brain of *P*. *argus*: Ionotropic Receptors (IRs), which are related to ionotropic glutamate receptors and found in all protostomes including crustaceans; Gustatory Receptors (GRs), which are ionotropic receptors that are abundantly expressed in insects but more restricted in crustaceans; and Transient Receptor Potential (TRP) channels, a diverse set of sensor-channels that include several chemosensors in diverse animals. We identified 108 IRs, one GR, and 18 homologues representing all seven subfamilies of TRP channels. The number of IRs expressed in the LF is far greater than in dactyls, possibly reflecting the contribution of receptor proteins associated with the ORNs beyond those associated with CRNs. We found co-receptor IRs (IR8a, IR25a, IR76b, IR93a) and conserved IRs (IR21a, IR40a) in addition to the numerous divergent IRs in the LF, dactyl, and brain. Immunocytochemistry showed that IR25a is expressed in ORNs, CRNs, and a specific type of cell located in the brain near the olfactory lobes. While the function of IRs, TRP channels, and the GR was not explored, our results suggest that *P*. *argus* has an abundance of diverse putative chemoreceptor proteins that it may use in chemoreception.

## Introduction

Acquiring environmental cues is key to the survival of animals, since it informs them about the location and quality of food, mates, predators, shelter, and other resources and risks. The first steps in detecting and discriminating environmental chemicals are performed by chemoreceptor cells, which possess the receptor proteins that bind the chemicals. Characterizing these receptor proteins is fundamental to understanding chemosensory transduction and, more broadly, mechanisms of chemical sensing.

**Fig 1:**
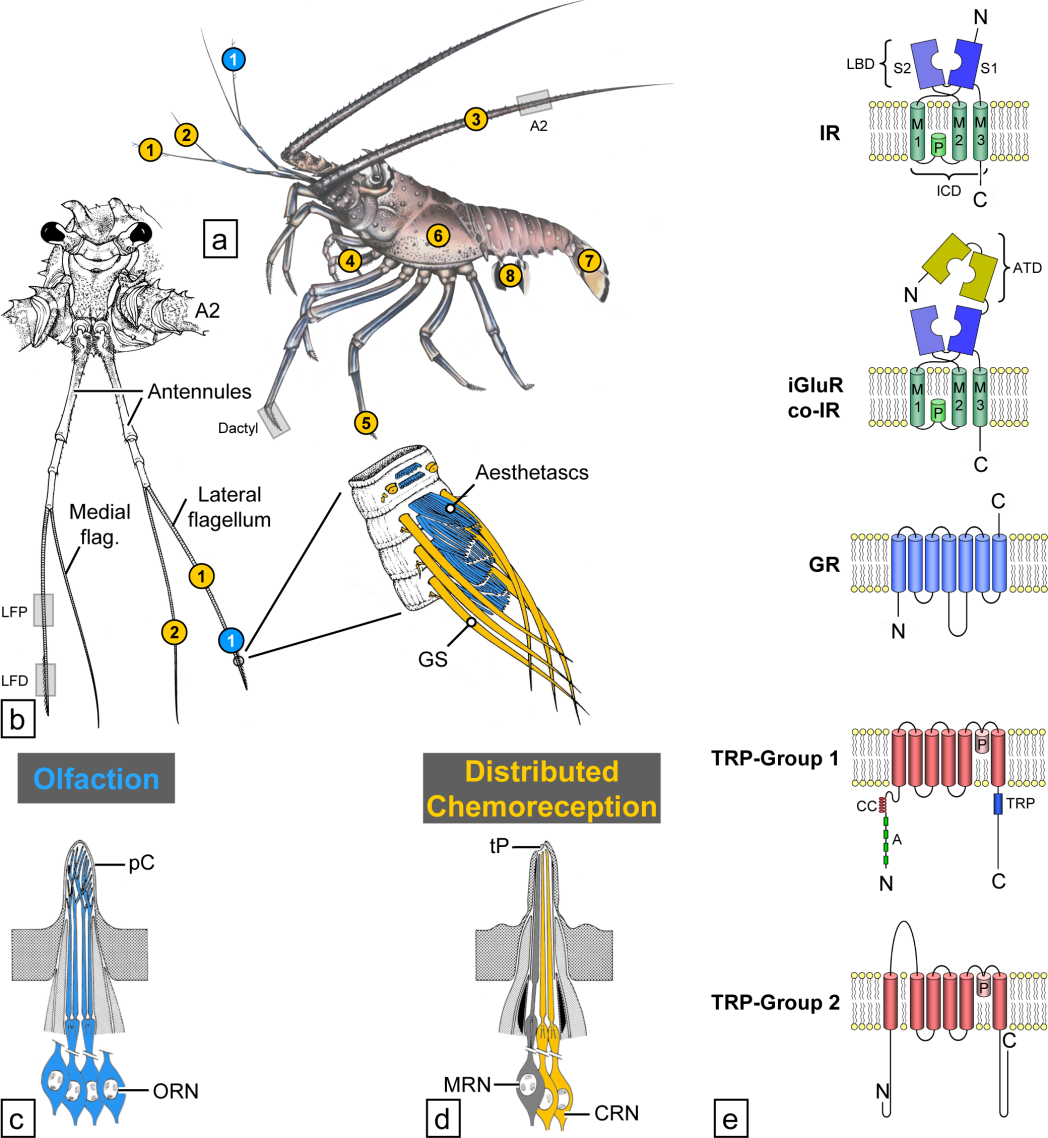
Overview of spiny lobster chemosensory systems. Modified from Derby et al. (2016) [9]. **(a)** Location of aesthetascs mediating olfaction (blue dots) and bimodal chemo- and mechanosensory sensilla mediating distributed chemoreception (yellow dots) on different body parts and appendages of *P*. *argus* (1—lateral flagellum of antennule, 2—medial flagellum of antennule, 3—second antenna, 4—mouthpart appendages, 5—walking legs, 6—gill chamber, 7—tail fan, 8—pleopods). Location of pieces of appendages used for immunocytochemistry and PCR indicated by gray boxes: dactyl, 2^nd^ antenna (A2). **(b)** Location of aesthetascs and bimodal chemo- and mechanosensory sensilla on the antennules. Aesthetascs (blue) are restricted to a tuft of sensilla on the distal third of the lateral flagellum. Bimodal chemo- and mechanosensory sensilla (yellow) among them, guard setae (GS) are associated with the aesthetascs but also occur on the proximal part of the lateral flagellum and on the entire medial flagellum. Location of pieces of appendages used for immunocytochemistry and PCR indicated by gray boxes: lateral flagellum of antennule proximal (LFP), lateral flagellum of antennule distal (LFD). **(c)** Schematic drawing of the cellular organization of olfactory sensilla. Olfactory sensilla, called aesthetascs, are exclusively innervated by olfactory receptor neurons (ORN, blue). The outer dendritic segments of the ORNs (modified cilia) are highly branched and covered by extremely thin and permeable cuticle (pC). **(d)** Schematic drawing of the cellular organization of distributed chemosensilla. Distributed chemosensilla are bimodal chemo- and mechanosensory sensilla innervated by a few mechanoreceptor neurons (MRN) and several chemoreceptor neurons (CRN). Dendrites of CRNs are unbranched and extend to a terminal pore (tP) at the tip of the sensillum. **(e)** Schematic drawing of the molecular structure of chemoreceptor proteins (iGluRs and Co-IRs, IRs, GRs, and TRP channels). Transmembrane domains of iGluRs and IRs (M1 –M3), pore loop (P), ligand binding domains (S1, S2), amino terminal domain (ATD), coiled-coil domain (CC), ankyrin repeats (A), TRP domain (TRP).

Although crustaceans are one of the largest and most diverse animal taxa with nearly 70,000 extant species living in diverse environments [1, 2], relatively little is known about their chemoreceptor proteins. Given that crustaceans are well-established models of chemoreception [3–6], the lack of data on their receptor proteins has limited our understanding of the organization of their chemical senses at the cellular and molecular levels. Crustaceans, in particular decapod crustaceans such as Caribbean spiny lobster, *Panulirus argus*, have two major chemosensory systems–olfaction and distributed chemoreception–which differ in their peripheral and central organization [5–9] (Fig 1). Olfaction is mediated by aesthetasc sensilla on the distal end of the lateral flagella of the antennules (first antennae). Aesthetascs are unimodal, being innervated only by olfactory receptor neurons (ORNs). More than 300 ORNs can innervate a single aesthetasc. Distributed chemoreception includes gustation plus other chemical senses except for olfaction. The sensilla in the distributed chemoreception pathway are diverse in form and are found not only on the antennules but also on the second antennae, mouthparts, legs, and other parts of the body. Despite structural diversity, the distributed chemosensilla have a fundamental characteristic: they are bimodal, being innervated by chemoreceptor neurons (CRNs) and mechanoreceptor neurons (MRNs). Chemoreceptor cells in olfaction and distributed chemoreception also differ in their central projections. ORNs project to the brain’s olfactory lobes, which have a glomerular organization [5]. CRNs project to neuropils in the brain, subesophageal ganglion, and thoracic and abdominal ganglia, which have a somatotopic organization and also serve as local motor centers for the appendages providing sensory input to the respective neuropils [5, 7, 8].

Olfaction and distributed chemoreception have some overlapping chemosensitivity and function, though they also detect different chemicals and mediate different behaviors. For example, both olfactory and distributed chemoreceptors on the antennules detect some of the same food-related chemicals, including representatives of amino acids, amines, nucleotides, organic acids, and other molecules. The detection of these chemicals drives the orientation of animals toward the source of the chemicals (reviewed in [5, 6]). Distributed chemoreceptors on the legs and mouthparts detect these same food-related molecules, yet stimulation of them drives different behaviors such as grabbing and ingestion of food or rejection of food, and stimulation of distributed chemoreceptor neurons in asymmetric sensilla on the antennules mediates grooming behavior [10]. However, olfaction and distributed chemoreception differ in their sensitivity to intraspecific chemical cues. Olfaction is largely responsible for detecting waterborne conspecific chemicals such as sex pheromones, social cues, and alarm cues, and then mediating various behaviors driven by these cues [11, 12]. Despite these known differences in olfaction and distributed chemoreception in crustaceans, the molecular basis for differences at the receptor level is unknown.

A phylogenetic and evolutionary approach to the study of chemoreceptor proteins has revealed that animals have adopted many different types of proteins as chemoreceptors, including a diversity of ligand-gated ion channels and metabotropic receptors (reviews: [9, 13–17]). The chemoreceptor proteins used by Protostomia and Deuterostomia are largely different from each other [9]. Protostomes predominately use ionotropic receptors as chemoreceptors, though GPCRs have been shown to function in a few protostomes, most notably in the nematode *Caenorhabditis elegans* [18–20], the sea hare *Aplysia californica* [21, 22], and the crown-of-thorns starfish [23]. In this study, we investigate the major classes of chemoreceptor proteins in the Protostomia, including Ionotropic Receptors (IRs), gustatory receptors (GRs), and transient receptor potential (TRP) channels.

An ancestral type of ionotropic receptor present in all Protostomia, and not present in the Deuterostomia, evolved from ionotropic glutamate receptors (iGluRs) and are thus called Variant Ionotropic Receptors, or simply Ionotropic Receptors (IRs) [24–27]. The iGluRs form tetrameric ion channels, with each monomer consisting of four main domains [28–30]: an extracellular amino-terminal domain (ATD) involved in assembly of the heteromeric channel; an extracellular ligand binding domain (LBD) consisting of two half-domains (S1 and S2) to which L-glutamate, glycine, or serine agonists bind; an ion channel domain (ICD) that forms the ion channel, consisting of three transmembrane domains (M1, M2, M3) and a pore loop (P); and an intracellular carboxyl-termination domain (CTD) [29] (Fig 1).

There are several families of IRs, including co-receptor IRs, conserved IRs, and divergent IRs. Co-receptor IRs–IR8a, IR25a, IR76b, and IR93a –are co-expressed with other IRs in cells and are necessary for the function of those receptor-channels. Out of these, IR25a and IR8a are the IRs most closely related to iGluRs in that they possess all four domains of the iGluRs. With the exception of IR25a and IR8a, IRs contain only three of four domains of iGluRs, either lacking or having a truncated ATD region (Fig 1). The other families of IRs are the conserved IRs and divergent IRs. Conserved IRs are IRs present across insects, crustaceans, and in the case of the co-receptor IRs IR25a and IR8a, all protostomes examined so far [24, 25, 27, 31]. Conserved IRs include IR21a, IR31a, IR40a, IR41a, IR60a, IR64a, IR68a, IR75, IR76a, IR84a, and IR92a. Due to the presence of their homologues in other species, all four co-receptor IRs are also considered to be conserved IRs. However, divergent IRs are species-specific IRs with no known homologues. When co-expressed with co-receptor IRs, the conserved and divergent IRs form functional heteromeric channels with ligand-specific binding properties that depend on the specific divergent IR that is expressed [32–35].

The number of IRs varies across species ranging from three in *C*. *elegans*, around 60 IRs in drosophilids, to over 120 in the termite *Zootermopsis nevadensis* [24, 25], and other protostomes have more or less than these. Though IRs are best studied in Insecta, they have also been identified in other arthropods, including myriapods (centipedes: [36]; millipedes: [37]), chelicerates (ticks: [27, 38]), and crustaceans [25, 31] (reviewed in [9], and see below). The genome of the water flea, *Daphnia pulex*, has 85 IRs, though the anatomical location of these IRs is unknown [25, 27, 31]. Some co-receptor IRs have been identified in a number of crustaceans including clawed lobsters, *Homarus americanus* [39], spiny lobsters [40, 41], hermit crabs [42, 43], seven species of shrimp [44], and copepods [31, 45–47]. The co-receptor IR25a is expressed in ORNs of the antennular lateral flagellum of *H*. *americanus* [39, 48], spiny lobster *P*. *argus* [40, 41], and hermit crab *Coenobita clypeatus* [43], and it also appears to be expressed in other chemosensory organs [41, 44]. However, very little is known about IRs, especially the divergent IRs, in most crustaceans. Complete sequences of two divergent IRs (and additional partial sequences) were identified in *P*. *argus* [41], 16 divergent IRs were found in the hermit crabs *Pagurus bernhardus* [42], and 22 divergent IRs were identified in *C*. *clypeatus* several of which were shown to be expressed in ORNs [43].

A major type of ionotropic chemoreceptor protein in Protostomia, apparently first appearing in insects and constituting a major class of receptors in them, is the Olfactory Receptors (ORs) [14, 15, 49–51]. These ionotropic ORs are not homologous with the GPCR ORs of deuterostomes and are evolutionarily related to arthropod Gustatory Receptors (GRs) (Fig 1) [13, 38, 49, 52–54] which are considered a more early-diverging class of ionotropic receptors. GR homologues ancestral to Arthropoda, for which chemosensory function has not been identified, are called Gustatory Receptor-Like proteins (GRLs). GRLs appeared early in metazoan evolution, at least in cnidarians and placozoans [13, 14, 31, 51].

To date, despite efforts, GRs have rarely been identified in crustaceans and ORs not at all. *D*. *pulex* has 58 GRs but no ORs [13, 14, 51, 55], and their anatomical location or involvement in chemical sensing has not been demonstrated. Eyun et al. (2017) [31] found a few GRs in some species of Copepoda and in a barnacle (Cirripedia). From their analysis, they concluded that GRs appeared early in metazoan evolution but expanded only in some groups: in the Arthropoda, the expansion was in Insecta and some Chelicerata but not most Crustacea.

Another class of receptors that are less explored in crustaceans is the transient receptor potential (TRP) channel superfamily. All TRP channels have six transmembrane regions and are cation channels. Based on sequence homology, TRP channels are classified into seven subfamilies belonging to two groups: group 1 includes subfamilies TRPA, TRPC, TRPM, TRPN, and TRPV; and group 2 includes subfamilies TRPML and TRPP (Fig 1) [56, 57]. TRP channels can be activated by several mechanisms including sensory stimuli such as temperature, light, chemicals, sound, and touch. Members of group 1 have been shown to be a part of chemosensory systems across animals including insects and nematodes. OSM-9 is a TRPV channel in *C*. *elegans* that mediates avoidance to bitter chemicals [58, 59]. TRPA1 is expressed in the labellum and mouthparts of *D*. *melanogaster* and detects aversive chemicals. TRPA1 also indirectly and directly mediates avoidance of citronella in flies and mosquitoes, respectively [60, 61]. *painless*, a member of the TRPA sub-family, is a chemoreceptor that prevents male-male courtship and also mediates avoidance of deterrent compounds [62]. Members of TRPC, TRP, and TRPL subfamilies are expressed in CO_2_-detecting ORNs in *D*. *melanogaster* and contribute to CO_2_ avoidance [56, 57].

Although their function has not been described in any crustacean, TRP channels are found in the few crustaceans examined. For example, *D*. *pulex* has 14 TRP channels representing all the subfamilies [63]. Among the decapods, *H*. *americanus* has a combined eight TRP channels in the transcriptomes of its central nervous system (CNS) and heart [64, 65], the Jonah crab, *Cancer borealis*, has six TRP channels in its CNS transcriptome [64], and the hermit crab, *C*. *clypeatus*, has one potential TRP channel, a homologue of the *D*. *melanogaster* TRPN channel, NompC, in its antennule transcriptome. However, TRP channels were not detected in the antennule transcriptome of the marine hermit crab, *P*. *bernhardus* [42].

The goal of our study was to use transcriptomics to identify chemoreceptor proteins in the olfactory and distributed chemoreception systems of a major crustacean model of chemoreception, the Caribbean spiny lobster *Panulirus argus*, and to compare them to homologous chemoreceptor proteins in *Drosophila*, *Daphnia*, and other species in major animal groups. We aimed to determine the number and diversity of receptor proteins of different types in olfactory sensilla bearing tissue (antennular lateral flagellum) and distributed chemoreception (leg dactyl) including whether these chemosensory organs express the same or overlapping sets of receptor proteins. We also examined the brain of *P*. *argus* due to the identification of chemoreceptor proteins, IR68a and IR75u, in the brain of the honey bee, *Apis mellifera* [25]. To accomplish these goals, we performed next-generation sequencing of mRNA preparations of the antennular lateral flagellum, leg dactyl, and brain, assembled their transcriptome, and performed bioinformatics searches for molecules of interest. In a very conservative estimate, we identified 108 IRs, with more of these expressed in the antennular lateral flagellum than in the dactyl. Using immunocytochemistry, we showed that IR25a is expressed in all ORNs and most CRNs but not in MRNs. We found IR25a expressed in specific cells of the ORN axon sorting zone near the olfactory lobe in the brain. We also identified one GR and homologues from seven subfamilies of TRP channels. Our results show a diversity of putative chemoreceptor proteins in *P*. *argus*.

## Materials and methods

### Animals

Experiments were performed on male and female Caribbean spiny lobsters, *Panulirus argus*. Most animals were obtained from the Florida Keys Marine Laboratory, and some animals were kindly provided by Dr. Don Behringer (University of Florida). Animals were held at Georgia State University in communal 800-L aquaria or in individual 10-L aquaria containing aerated, recirculated, filtered artificial seawater (Instant Ocean, Aquarium Systems, Mentor, OH) in a 12-hr:12-hr light:dark cycle. They were fed shrimp or squid three times per week.

### Tissue collection and RNA isolation for generating transcriptomes

Tissue was collected from four animals: female 1 (F1) with carapace length 66 mm, weight 255 g; female 2 (F2) with carapace length 80 mm, weight 397 g; female 3 (F3) with carapace length 63 mm and weight 232 g; and male 1 (M1) with carapace length 77 mm and weight 364 g. Tissues were dissected from animals that were anesthetized in ice. Three tissues were collected, shown in Fig 1. The aesthetasc-bearing region of both antennular lateral flagella (LF) of F1 and M1 was collected and pooled. The dactyl of the right second walking leg (dactyl) leg was collected from F2. The supraesophageal ganglion, or brain, was collected from F3. For collecting LF and dactyl, the soft tissue within the cuticle was dissected out. For brain, the head was separated from the rest of the body and the brain was removed from the posterior aspect, with care being taken to prevent hepatopancreas from entering the head space during dissection. Immediately following dissection, collected tissues were instantly frozen in liquid nitrogen and stored at -80°C.

Total RNA was extracted from these tissues using Tri-Reagent (Sigma-Aldrich, St. Louis, Missouri). Frozen tissues were weighed and then homogenized in Tri-Reagent using sterile disposable pestles. Chloroform was used for separation through centrifugation, and RNA was precipitated with ethanol. To ensure the quality of RNA and to remove any contamination with DNA, protein, or carbohydrate, the precipitated RNA was reconstituted in diethylpyrocarbonate (DEPC)-treated water and again precipitated using lithium chloride. Next, the RNA was reconstituted in DEPC-treated water, and other contaminants such as sodium dodecyl sulfate (SDS) and SDS-bound proteins were precipitated out of the RNA using potassium acetate. Total RNA was then precipitated out of solution with ethanol and reconstituted in DEPC-treated water. The total RNA extracted for each tissue was quantified and checked for purity using NanoDrop 2000c spectrophotometer (Thermo Fisher Scientific, Waltham, Massachusetts). Running agarose gel electrophoresis and staining the gels with ethidium bromide visualized RNA integrity. Aliquots of the total RNA extracted for each tissue were frozen over liquid nitrogen and stored at -80°C.

### Illumina sequencing, *de novo* assembly, and transcript abundance estimation

Total RNA extract for each tissue was diluted to 100 ng/μL in TE buffer (10 mM Tris, 1 mM EDTA, pH 7.5). Diluted RNA samples were instantly frozen in liquid nitrogen and shipped on dry ice to Beckman Coulter Genomics for quality assessment on Agilent Bioanalyzer2000 and TapeStation, mRNA specific cDNA synthesis, and paired-end sequencing of cDNA on Illumina HiSeq 2500 high-throughput sequencer. The read length was 2x100 (base pair reads) for LF and dactyl and 2x125 (base pair reads) for brain, and the number of reads per sample was > 120 million. Prior to data delivery, adapter sequences incorporated for tracking Illumina reads from multiplexed samples were removed.

All high performance computing (HPC) was performed on ORION and ACoRE HPC systems at Georgia State University [66]. A transcriptome was generated by combining raw reads from the three tissues by following the *de novo* assembly protocol [67] for the program Trinity v2.6.5 [68] (S1 Text). Prior to assembly, reads were quality trimmed with default settings using the Trimmomatic software [69] bundled into Trinity. The TransDecoder program (http://transdecoder.github.io/) was used to predict proteins with open-reading frames (ORFs) in each transcriptome. The predicted proteins for the transcriptome are referred to as ‘Parg protein database.’

CD-Hit [70] was performed on the transcriptome to remove redundancy. BUSCO v3 [71, 72] was run on the transcriptome before and after running CD-Hit to analyze the completeness of the assembly (S2 Text). Following removal of redundancy, the abundance of transcripts was estimated using RSEM, an alignment-based quantification software [73]. Custom ‘R’ scripts were used to extract the counts for each tissue for the transcripts of interest from the gene counts matrices that were generated using RSEM perl scripts bundled in Trinity, and were plotted using the ‘heatmap.2’ function in R (S1 and S2 Figs, S2 and S3 Tables).

### IR identification, sequence alignment, and phylogenetic analysis

iGluR and IR protein sequences from the common fruit fly (*Drosophila melanogaster*, Dmel), mosquito (*Anopheles gambiae*, Agam), water flea (*Daphnia pulex*, Dpul), and Caribbean hermit crab (*Coenobita clypeatus*, Ccly) were collected from published data [24, 25, 41, 43] and NCBI databases. These were used as query sequences to screen for iGluRs and IRs in our Parg transcriptome and protein databases. NCBI-BLAST+ versions 2.3+ and 2.4.0 on ORION and ACoRE were used for tBLASTn and BLASTp searches on the Parg transcriptome and protein databases. Sequence hits with e-value < e^-4^ from the tBLASTn and BLASTp searches, along with Dmel and Dpul query sequences, were used as queries for PSI-BLAST searches against the Parg protein databases. These hits were then further screened for having both the ICD and LBD (Fig 1), which characterize iGluRs and IRs [25]. This screening was performed with TMHMM v2.0 [74] for transmembrane domain prediction and signature-screened against InterPro [75] with InterProScan 5 (v5.28–67.0) [76] for conserved Pfam domains. The ICD domain and S2 of the LBD were predicted by the presence of the Pfam domain, PF00060 (which contains M1, P, M2, S2, and M3, see Fig 1). S1 of the LBD was predicted by the presence of the Pfam domain, PF10613 [25, 77].

To perform phylogenetic analysis, we used predicted protein sequences for putative IRs and iGluRs from the Parg protein databases containing both PF00060 and PF10613 domains (S3 and S4 Texts) and reference sequences of IRs and iGluRs from Dmel and Dpul. These sequences were aligned with MAFFT [78, 79] using default settings and visualized on Jalview [80]. Alignments were manually trimmed to remove gaps (S5–S10 Texts) guided by amino acid conservation as annotated on Jalview. Sequences with < 150 amino acid residues that were missing large regions of the S1 or S2 domains of the LBD and ICD were removed from further analysis, with some exceptions. The best model of substitution was identified using ModelFinder [81], and maximum likelihood phylogenetic trees were constructed using IQ-Tree [82, 83]. Confidence values for the trees were generated using ultrafast bootstrap (UFBoot) [84] integrated into IQ-Tree. The phylogenetic trees were visualized using FigTree v1.4.2 (http://tree.bio.ed.ac.uk/software/figtree/), and color schemes were edited on Adobe Illustrator CS6, San Jose, CA.

Phylogenetic analysis of conserved IRs also included conserved IR sequences from several insect species: *Aedes aegypti* (Aaeg), *Culex quinquefasciatus* (Cqui), *Anopheles gambiae* (Agam), *Bombyx mori* (Bmor), *Tribolium castaneum* (Tcas), *Apis mellifera* (Amel), *Nasonia vitripennis* (Nvit), *Acrythosiphon pisum* (Apis), and *Pediculus humanus humanus* (Phum); and two gastropod molluscs: *Aplysia californica* (Acal) and *Lottia gigantea* (Lgig).

### IR and iGluR nomenclature

IRs were named with a prefix of the species ‘Parg-’ and a random number assignment starting from 1000 and chronologically increasing. For example, the IRs were named PargIR1000, PargIR1001, and so on. Homologous sequences are named according to their IR homologues; e.g., PargIR25a, PargIR8a, and so on. NMDA-like iGluRs were named according to their homologues from Dmel and Dpul. Other iGluRs were named with random numbers.

### GR identification and sequence alignment

GR sequences from Dmel, Dpul, and a copepod (*Eurytemora affinis*, Eaff) were used as queries in BLASTp searches of the Parg transcriptome. All hits against the query sequences were selected for further analysis even though their e-values were > e^-4^. This was expected based on the high sequence divergence among GRs of other species [14, 51, 55]. The transcriptome was also screened using InterProScan for the Pfam domain family, 7tm_7 (PF08395), since this family includes GRs and ORs from insects.

### TRP channel identification and sequence alignment

TRP channel sequences from group 1 and group 2 subfamilies of Dmel, Dpul, Jonah crab (*Cancer borealis*, Cbor), American clawed lobster (*Homarus americanus*, Hame), nematode (*Caenorhabditis elegans*, Cele), mouse (*Mus musculus*, Mmus), and rat (*Rattus norvegicus*, Rnor) were used as queries in BLAST searches against the Parg transcriptome and protein database. TRP channel sequences in Dmel were extracted from www.flybase.org. TRP channel sequences from other insects (Bmor, Tcas, Amel, Nvit, Phum) were collected from Matsuura et al. (2009) [85]. Cele and mammalian sequences were extracted from publicly available databases on NCBI. TRP channel sequences of Dpul [63] were extracted from the JGI genome portal (https://genome.jgi.doe.gov/Dappu1/Dappu1.home.html). The rest were collected from NCBI databases. Only those sequences from the Parg transcriptome and protein database with e-values < e^-4^ to the queries were selected for further analysis. In parallel, we also screened the Parg transcriptome with InterProScan for TRP channel domain regions of different subfamilies. These included Pfam groups PF06011, PF08344, PF00520, PF12796, PF00023, PF16519, and PF08016, which are TRP, TRP_2, ion transport, ankyrin 2, ankyrin, TRPM_tetra, and PKD channel domains, respectively. For maximum likelihood phylogenetic analyses, along with the query sequences, the target sequences were aligned with MAFFT (S13–S15 Texts), and maximum likelihood phylogenetic trees were constructed using IQ-Tree after ModelFinder assessed the best model of substitution as described above.

### PCR

For PCR, total RNA was extracted from the following tissues of a male *P*. *argus* (549 g, 86.4 mm carapace length) after anesthetizing the animal on ice for about 20 min: (1) the distal, aesthetasc-bearing part of the LF (LFD); (2) the proximal, non-aesthetasc bearing part of the LF (LFP) (note that LFP lacks the 20 proximal-most annuli in the non-aesthetasc region, which contains the proximal proliferation zone and its developing aesthetascs [86]); (3) flagella of second antennae (A2); (4) dactyls of second pereiopods (walking legs) (dactyl); (5) central brain (brain); and (6) green glands (GG). After anesthetizing the animal on ice for about 20 min, appendages and head were cut off. All appendages were wiped with 100% ethanol to remove attached epibionts before they and the remaining body parts were dissected further under autoclaved *P*. *argus* saline. From LF, A2, and dactyls, the internal tissue including the epithelium was removed from the surrounding cuticle. All tissues were instantly frozen in liquid nitrogen and stored at -80° C.

For extraction of total RNA, the appropriate amount of TRIzol (Thermo Fisher Scientific, Waltham, Massachusetts) was added to tissue samples (10 μl of TRIzol per mg of sample) and these were homogenized using disposable tissue grinders (Biomasher II, Kimble Chase, Vineland, New Jersey) or sterilized glass tissue homogenizers. Extracts were processed according to the TRIzol protocol until phase separation, and then RNA was purified using the Direct-zol RNA Mini Prep (Zymo Research, Irvine, California) including treatment with DNAse I to remove genomic DNA. About 1 μg of total RNA was used for cDNA synthesis with SuperScript III reverse transcriptase (Thermo Fisher Scientific—Invitrogen, Waltham, Massachusetts) and random hexamers following the manufacturer’s instructions.

Primers for PCR (S1 Table) targeting the housekeeping gene GAPDH and the genes of interest from the Parg transcriptome (NMDA-R1, IR25a, IR8a, IR93a, IR1028, IR1074) were designed using Primer Blast (NCBI) and purchased from Integrated DNA Technologies (Coralville, Iowa). PCR amplification was performed in 50 μl reactions containing about 250 ng of cDNA with the DNA polymerase Phusion according to the manufacturer’s instructions (Thermo Fisher Scientific, Waltham, Massachusetts) using the thermal cyclers Mastercycler SXI (Eppendorf, AG, Hamburg, Germany) and C1000 Touch (Bio-Rad Laboratories, Hercules, California). Annealing temperatures of primer pairs were calculated with an online Tm calculator (New England Biolabs, Ipswich, Massachusetts). PCR products were subjected to gel electrophoresis (1.5% agarose gel) together with a 100 bp DNA ladder (G-Biosciences, St. Louis, Missouri) for size determination.

To determine if PCR products matched the target sequences, bands of the expected size were cut from the agarose gel and cDNA was extracted from them using the PureLink kit (Thermo Fisher Scientific—Invitrogen, Waltham, Massachusetts). Extracted cDNA was sequenced in the Georgia State University (GSU) molecular core facility.

### Immunocytochemistry

For immunocytochemistry, LF, dactyls of pereiopods, second antennae, and brains of male and female *P*. *argus* (34–65 mm carapace length and 45–250 g in weight, n = 6) were dissected after anesthetizing the animals on ice for about 20 min and were fixed for 6–24 hr at room temperature by immersion in 4% paraformaldehyde in 0.1 M Sörensen phosphate buffer (SPB) containing 15% sucrose. Prior to incubation in fixative, LF were cut into 8-annuli long pieces (covering the LFD and LFP) as described previously [87], dactyls were cut into 2 or 3 pieces, and second antennae were cut into 5–8 annuli long pieces with coarse scissors. After fixation, LF, dactyls, and second antennae were decalcified by incubation in 10% EDTA (in SPB) for about one week (with several changes of the medium). All fixed tissues were stored in 0.02 M SPB with 0.02% sodium azide at 4°C until sectioning. For sectioning, tissues were embedded in gelatin (100 bloom for brain, 300 bloom for LF, dactyls, and second antennae) and cut on a vibrating microtome (VT 1000 S; Leica, Wetzlar, Germany) into 80–100 μm thick sections as described in detail previously for brain [88] and LF [87]. LF, dactyls, and second antennae were cut sagittally and brains were cut horizontally or sagitally (after splitting them into hemibrains).

Free-floating sections were incubated overnight at room temperature with an affinity-purified polyclonal rabbit antiserum against IR25a of the American lobster, *H*. *americanus* (anti-HaIR25a - courtesy of Dr. Tim McClintock, University of Kentucky) diluted 1:750 in SPB containing 0.3% Triton-X-100 (TSPB). Anti-HaIR25a (previously annotated as anti-GluR1) was generated using two non-overlapping peptides (P1: TGEGFDIAPVANPW; P2: REYPTNDVDKTNFN) from the C-terminus of *H*. *americanus* IR25a (originally annotated as OET-07; Genbank accession #AY098942) [39, 48]. Sequence alignments of P1 and P2 with the deduced amino acid sequences of all IRs and iGluRs of *P*. *argus* identified in our transcriptome sequencing project showed matches for both peptides only in *P*. *argus* IR25a (P1: 79% identity; P2: 86% identity). This high degree of antigen identity between *H*. *americanus* and *P*. *argus* strongly suggests that anti-HaIR25a specifically labels IR25a in *P*. *argus*, as has been demonstrated by Western blots for *H*. *americanus* [48].

For sections from LF, dactyls, second antennae, and some brains, anti-HaIR25a was combined with a mixture of two mouse monoclonal antibodies against modified α-tubulin isoforms that are enriched in neurons [89] to achieve labeling of all sensory neurons. These tubulin antibodies were anti-tyrosine tubulin (T9028, clone TUB-1A2, Sigma-Aldrich, St. Louis, Missouri) diluted 1:2000 and anti-acetylated tubulin (sc-23950, Santa Cruz Biotechnology, Dallas, Texas) diluted 1:200. After incubation in primary antibodies, sections were rinsed 4 x 30 min in TSPB and then incubated in a mixture of two secondary antibodies, goat anti-rabbit CY3 (111-165-003, Jackson Immunoresearch, West Grove, Pennsylvania) diluted 1:400 and goat anti-mouse DyLight-488 (35502, Thermo Fisher Scientific, Waltham, Massachusetts) diluted 1:100 in TSPB. After rinsing 3 x 30 min in TSPB, sections were incubated for 20 min in Hoechst 33258 diluted 1:150 in TSPB from a stock solution of 1 mg/ml to stain nuclei. After a final rinse in SPB, sections were mounted on slides in 1:1 glycerol:SPB containing 5% DABCO (diazabicyclol[2.2.2]octane) to prevent photobleaching. Coverslips were secured with nail polish, and slides were stored at 4° C or at -20° C (for extended storage time). In some brains, labeling with anti-HaIR25a was combined with labeling by the lectin wheat germ agglutinin (WGA) that we previously identified as selective neuronal marker for the brain of *P*. *argus* [90]. In these brain sections, WGA-AlexaFluor-488 (Thermo Fisher Scientific—Invitrogen) diluted 1:1000 replaced goat anti-mouse DyLight-488 in the secondary antibody incubation medium.

Labeled sections were viewed and imaged at low magnification in an epifluorescence microscope equipped with color CCD camera (AxioScope FL LED with Axiocam 503, Carl Zeiss Microscopy, Thornwood, New York) and imaged at higher magnification in a confocal microscope (LSM 700, Carl Zeiss Microscopy) using the associated software package ZEN. Stacks of 0.3- to 1.0-μm thick optical sections covering the entire section thickness of 60–80 μm were collected. A different software package (LSM Image Browser Version 4.2.0.121, Carl Zeiss MicroImaging GmbH, Jena, Germany) was used to select sub-stacks of optical sections and collapse them to two-dimensional images using maximum-intensity projection.

### Histological staining with ethyl gallate and methylene blue

Brains with attached antennular nerve roots were perfusion-fixed with 5% glutaraldehyde in 0.1 M SPB + 15% sucrose for 4 hr and post-fixed for 2–3 hr with OsO4 according to a TEM-fixation protocol [91]. After dehydration in an ascending ethanol series and incubation in propylene oxide (2 x 30 min), brains were either embedded in hard Epon for semi-thin (1–2 μm) sectioning and subsequent staining with methylene blue (1% in aqueous borax) or were incubated in ethyl gallate solution, embedded in Spurr’s resin, and cut into 10–20 μm thick sections (according to methods described in detail in Schmidt et al. (1992) [92]). Sections were viewed and imaged in a bright field microscope equipped with color CCD camera (AxioScope FL LED with Axiocam 503, Carl Zeiss Microscopy, Thornwood, New York).

### Scanning electron microscopy

Walking leg dactyls were cut off, cleaned by sonication for about 10 min (VWR Model 50T, VWR International, West Chester, Pennsylvania), cut into 2 or 3 pieces, and were fixed by immersion in 5% glutaraldehyde in 0.1 M SPB + 15% sucrose for 4 hr and post-fixed for 2–3 hr with OsO4 according to a TEM-fixation protocol [91]. Fixed pieces were dehydrated, air-dried from hexamethyldisilazane, sputtered with palladium and viewed in a scanning electron microscope equipped with digital image capturing (Stereoscan 420 with LEO-32, Leica, Wetzlar, Germany) as described in detail by Schmidt and Derby (2005)[10].

### Processing of digital images

The digital images were processed by filtering out high frequency noise and by adjusting brightness and contrast with a free image and photo editing software (Paint.net 4.0.16, dotPDN LLC) before they were arranged into the final figures with Adobe Illustrator CS6.

## Results

### Identification of iGluRs and IRs in *P*. *argus*

To identify iGluRs and IRs in *P*. *argus*, we probed the predicted protein sequences (Parg protein database) from the transcriptome generated from three tissues–distal lateral flagellum of first antennule (LF), walking leg dactyls, and brain. We conducted BLAST searches with iGluR and IR sequences from *Drosophila melanogaster* (Dmel) and *Daphnia pulex* (Dpul) as query files and also performed an InterProScan screen of the transcriptomes for conserved the Pfam domains, PF00060 (consisting of M1, P, M2, S2, and M3 regions of iGluRs and IRs) (S3 Text) and PF10613 (consisting of S1 region of iGluRs and IRs) (S4 Text). The results of the InterProScan for the number of “Trinity genes” representing unique Trinity ID numbers and therefore protein sequences for iGluRs and IRs [68] yielded 342 sequences with PF00060 domain, 286 sequences with the PF10613 domain, and 132 with both of these domains.

**Fig 2:**
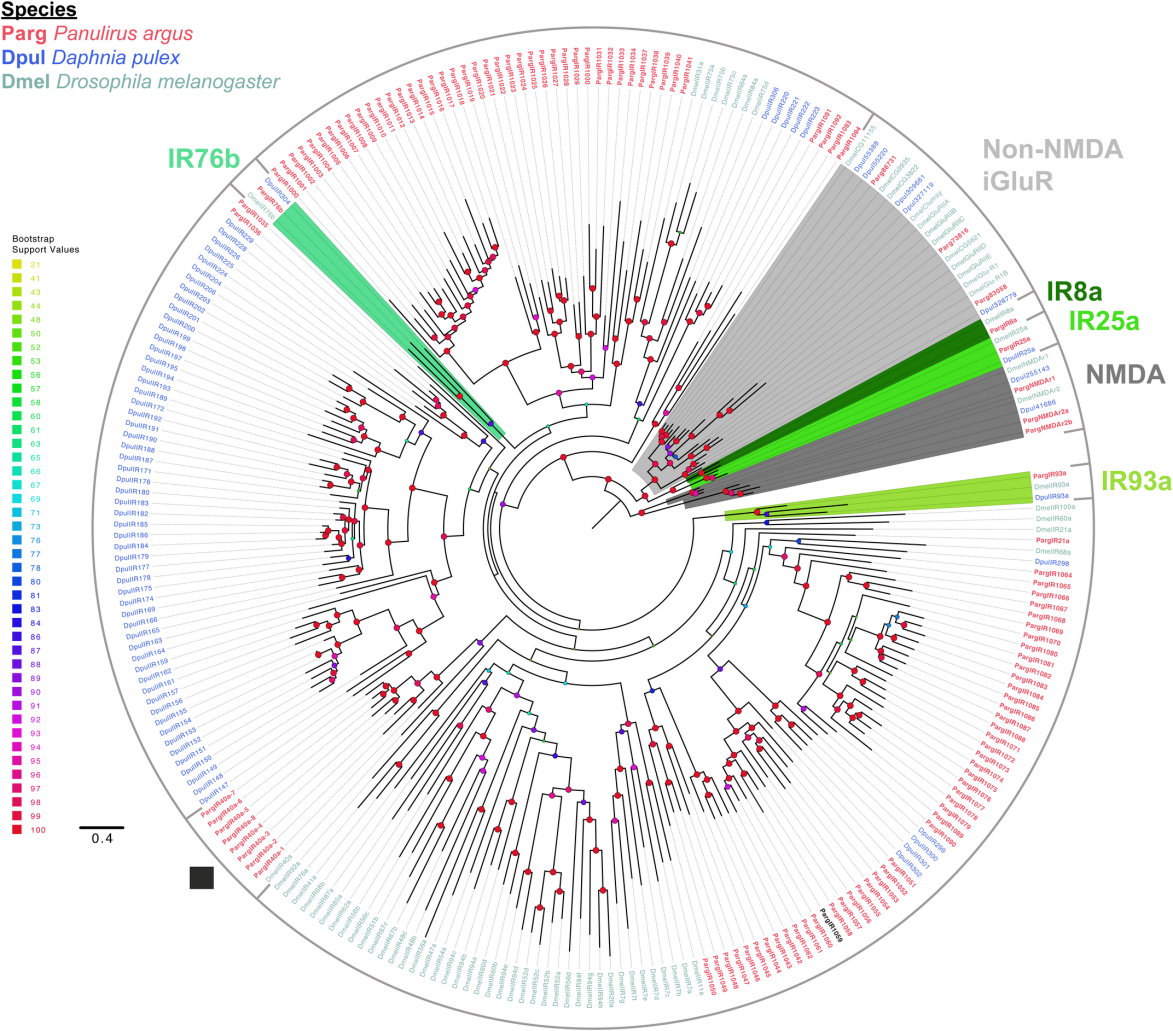
Maximum likelihood phylogenetic tree of iGluRs and IRs. Colored areas of the tree represent iGluRs (shades of grey) and co-receptor IRs (shades of green). ■ indicates conserved IR sequences of the IR40a family. Sequence colored in black indicates the Parg divergent IR that has an amino acid substitution in the conserved glutamate binding site in the S1 region of the LBD. The tree was built under LG+F+G4 model of substitution with 1000 ultrafast bootstrap (UFBoot) replications and visualized on FigTree v1.4.2. Bootstrap values on some internal branches of divergent IRs are low due to incomplete sequences and high sequence divergence across the different species. The tree was unrooted but drawn with the NMDA clade as root. The scale bar represents expected number of substitutions per site.

To refine the identification of Parg homologues of iGluRs and IRs in the Parg transcriptome, we performed a phylogenetic analysis of all selected sequences (S5 Text) together with iGluRs and IRs from Dmel and Dpul. In this analysis, we used only the protein sequences having two domains of iGluRs and IRs (i.e., both PF00060 and PF10613 domains). Furthermore, sequences from this set that were short and had large gaps in these two domains, and sequences that did not have any transmembrane regions as predicted by TMHMM 2.0, were not included in the following analysis, with a few exceptions that included sequence hits to conserved IRs from the BLAST searches. Thus, this set of 114 selected sequences from the transcriptome represents a conservative estimate of IRs and iGluRs. With over 340 unique sequences in the Parg transcriptome having at least the PF00060 domain, we expect the total number of IRs to be greater in Parg than our current estimate. Based on our phylogenetic analyses, the selected sequences were categorized into four groups: iGluRs, co-receptor IRs, conserved IRs, and divergent IRs, and the results are shown in Fig 2, Table 1, and Supplementals S1 Fig and S2 Table.

**Table 1:** Number of unique iGluRs and IRs in *P*. *argus*, *D*. *pulex*, and *D*. *melanogaster*.

Species	iGluR	Co-receptor IR	Conserved IR	Divergent IR
***P*. *argus***	6	4	9	95
***D*. *pulex***	10	3	1	81
***D*. *melanogaster***	14	4	14	48

Only those Parg sequences that were selected for phylogenetic analyses are included here. The four groups of sequences are iGluRs (including NMDA and non-NMDA receptors), co-receptor IRs, conserved IRs, and divergent IRs. The numbers for *D*. *pulex* and *D*. *melanogaster* are acquired from sequenced genomes [25].

#### iGluRs

Six iGluRs were identified in the Parg transcriptome. They phylogenetically cluster with iGluRs of Dmel and Dpul, and as expected, are distinguished from IRs by lacking the highly divergent LBD of the IRs [24]. Parg iGluRs include homologues to the Dmel NMDA receptors NMDAr1 and NMDAr2, non-NMDA (kainate) receptors GluRII and DmelCG11155, and AMPA receptors DmelGlu-R1 and DmelGlu-R1b. One Parg sequence is homologous to Dmel NMDAr1 sequence. Parg also has two sequences that are homologous to the Dmel NMDAr2 sequence, PargNMDAr2a and PargNMDAr2b. Among the non-NMDA iGluRs, there are two homologues to kainate receptors, Parg86731 and Parg73816. There is one homologue to Dmel AMPA receptors, Parg83058.

#### IRs

Our analysis demonstrated 108 sequences to be IRs in the Parg transcriptome. These included four co-receptor IRs, nine conserved IRs, and 95 divergent IRs (Table 1).

**Co-receptor IRs.** Four co-receptor IRs were identified in the Parg transcriptome: IR8a, IR25a, IR76b, and IR93a. These sequences and their tissue specific expression are shown in Fig 2 and S1 Fig. IR25a and IR8a cluster close to the iGluRs because only they have a defined ATD like the iGluRs. We did not identify IR8a in the Dpul database, as others have also reported [25, 31].

**Divergent IRs.** The Parg transcriptome had 95 divergent IRs. Out of these 95 divergent IRs, four are putative IR75-like sequences (PargIR1091, PargIR1092, PargIR1093, and PargIR1094. See below in **Conserved IRs**) and one is a putative IR68a homologue (PargIR68a-put. See below in **Conserved IRs**). The distribution of the remaining divergent IRs between the tissues was highly skewed and mostly non-overlapping (S1 Fig and S2 Table): 51 divergent IRs were only in LF, two divergent IRs only in dactyl, and over 14 divergent IRs were found in both LF and dactyl. Two additional divergent IRs that are different from our 95 sequences were identified in the LF by Corey et al. (2013) [41], who named them IR4 and IR7.

**Fig 3:**
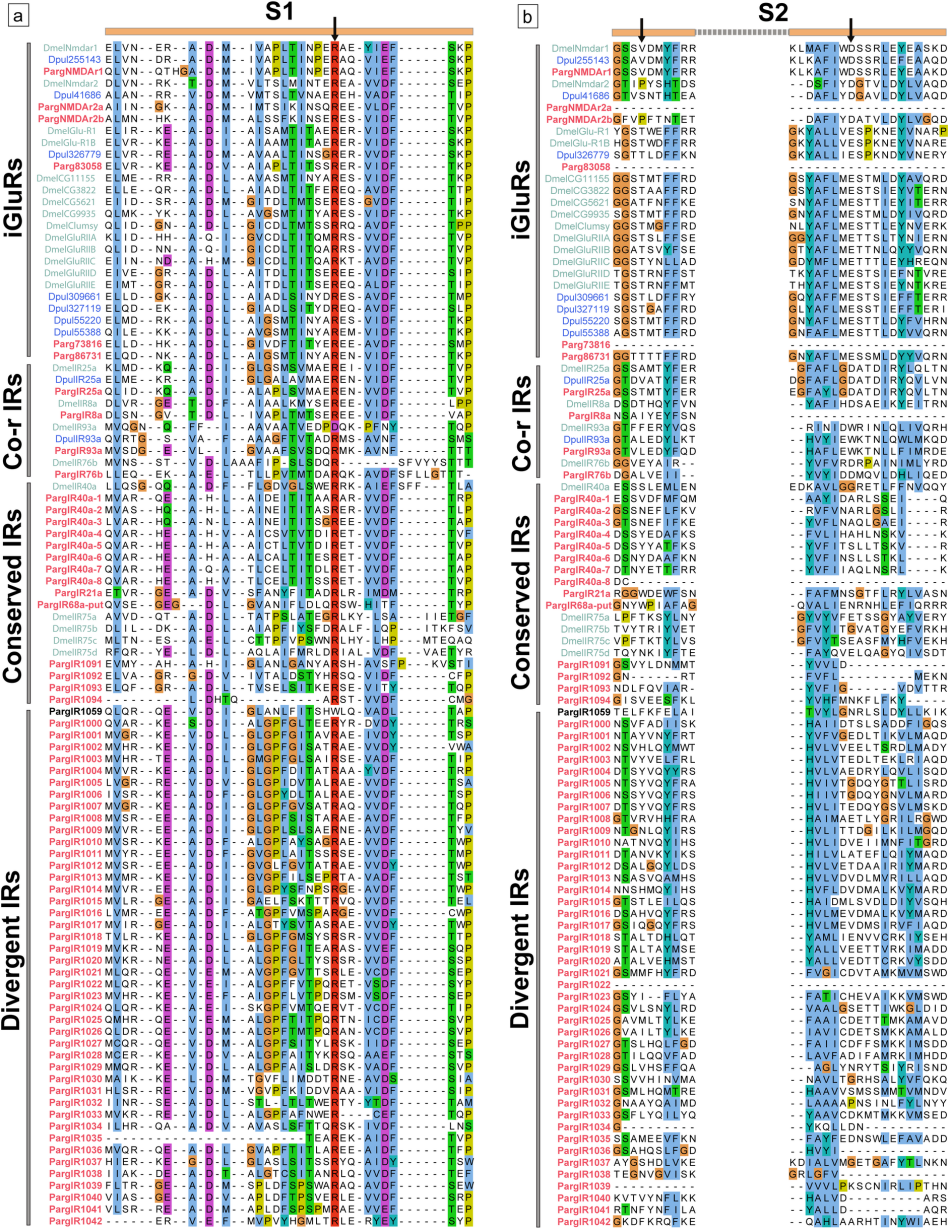
Multiple sequence alignment of iGluRs and IRs of Parg, Dmel, and Dpul. This analysis shows that the LBD is highly divergent within the IRs and in comparison to iGluRs. Parg (red), Dmel (mint), and Dpul (blue) sequences are organized based on sequence homology in Fig 2. Divergent IR, PargIR1059, an exception to ‘R’ conservation in S1 lobe, is indicated in black. Glutamate binding sites are indicated with arrows. **(a)** S1 lobe of LBD. **(b)** S2 lobe of LBD. The sequences were aligned with MAFFT and visualized on Jalview. The residues were colored according to the Clustal X color scheme on Jalview. Criteria for the color scheme: http://www.jalview.org/help/html/colourSchemes/clustal.html.

Multiple sequence alignments of iGluRs and IRs of Parg and Dmel illustrate two major similarities and one dissimilarity between these two species (Fig 3; S6 Text). The first similarity is a high variability of amino acids in S1 and S2 regions of the IRs of Parg and Dmel. This is probably related to the specificity of ligand binding of these receptors. The second similarity is that the S2 region of most IRs of both Parg and Dmel lack both the amino acid residues present in iGluRs that bind the glutamate ligand. The dissimilarity in the IRs of Parg and Dmel is that only 31% of the IRs in Dmel have the glutamate binding arginine residue (R) in S1 that is conserved in iGluRs [24, 29], but 99% (107 of 108) of the Parg IRs have this residue. One Parg divergent IRs, PargIR1059, lacks this arginine residue (Fig 2, sequence in black). Instead of the conserved ‘R’ residue of S1, PargIR1059 has a non-polar tryptophan (W) residue. PargIR93a does not have a substitution is this conserved site unlike Dmel IR93a (Fig 3; [24]).

**Fig 4:**
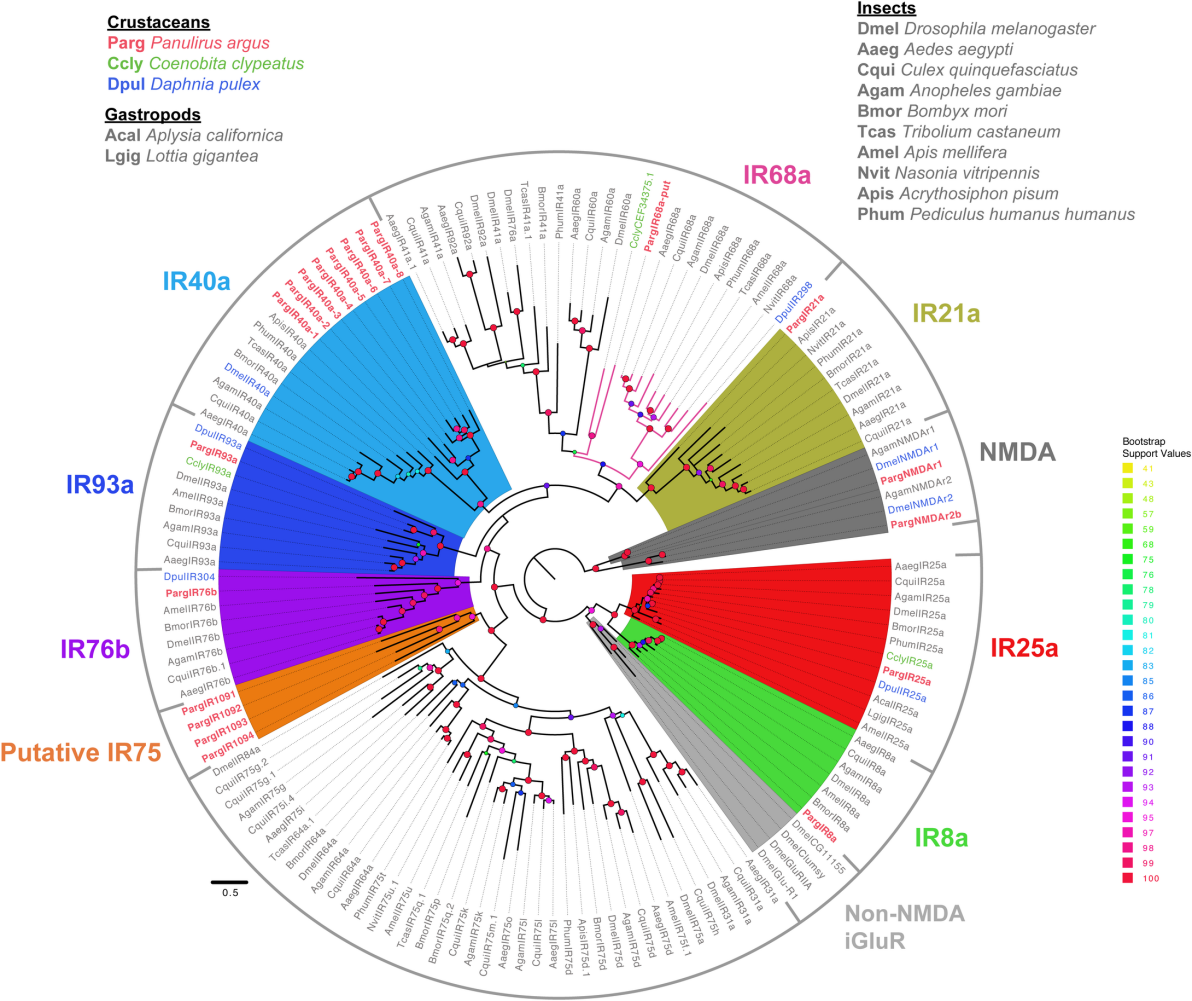
Maximum likelihood phylogenetic tree of homologous sequences of conserved IRs. Only conserved IR groups with Parg homologues are colored. Pink branches represent homologous sequences of IR68a with the Ccly sequence, the putative PargIR68a-put, and DpulIR298 as crustacean representatives. Sequences were aligned with MAFFT and visualized on Jalview for editing. The tree was built on IQ-Tree under LG+F+G4 model of substitution with 1000 UFBoot replications and visualized on FigTree v1.4.2. Bootstrap values in some internal nodes are low due to incomplete sequences. The tree was unrooted but drawn with the NMDA clade as the root. The scale bar represents the expected number of substitutions per site.

**Conserved IRs.** Based on BLAST search results and phylogenetic analyses (Figs 2 and 4), we identified nine homologues to conserved IRs in the Parg transcriptome. Out of these nine conserved IRs, one conserved IR is PargIR21a, while the other eight are an expanded family of IR40a homologues. These sequences are denoted as Parg IR40a-1 to Parg IR40a-8 (Fig 4).

To represent the Parg co-receptor IRs and conserved IRs in a broader phylogenetic context, we constructed another tree using only conserved IRs and with more species included. These include Parg, Dmel, and Dpul as in Fig 2, another decapod crustacean (Ccly), eight additional insect species (Aaeg, Cqui, Agam, Bmor, Tcas, Amel, Nvit, Apis, and Phum), and two gastropods (Acal and Lgig). The results from this analysis (Fig 4) highlight three major points. First, this analysis confirms the results of Fig 2 in showing four co-receptor IRs (IR8a, IR25a, IR76b, IR93a) and conserved IRs (IR21a and the expanded IR40a family) in the Parg transcriptomes in a broader phylogenetic context. Second, it also suggests that Parg may have additional conserved IRs, including members of the IR75 clade that are present in the transcriptome. There are four sequences that we consider putative conserved IRs due to their proximity to the insect IR75 clade of conserved IRs. The four Parg sequences (PargIR1091, PargIR1092, PargIR1093, PargIR1094) may be homologous to the sequences from other species in the IR75 clade (Figs 2 and 4). In this study, we classified these putative IR75 sequences as divergent IRs and the numbers are appropriately reflected in Table 1. Third, one Dpul IRs, DpulIR304, which was previously classified as divergent IR by Croset et al. (2010) [25], clustered with the homologues of the co-receptor IR76b. Another Dpul divergent IR, DpulIR298, may also be a conserved IR. Based on BLAST searches against non-redundant NCBI databases and our phylogenetic analyses, we classify it as homologue of IR68a. A Ccly hermit crab IR (CEF34375.1) clustered with insect IR68a [43], and we identified a putative IR68a in Parg, PargIR68a-put. We consider this sequence as a putative homologue due to the sequence being incomplete and reciprocal BLAST searches against non-redundant NCBI databases returning hits for other conserved insect IRs. We also did not find other insect conserved IRs in Parg or Dpul.

We point out that these analyses did not include over 200 sequences of putative Parg IRs due to their small sequence length and missing domain regions (S3 and S4 Texts), and therefore our estimates of the type and number of IRs are conservative.

#### Immunolocalization of the *P*. *argus* IR25a receptor

**Fig 5:**
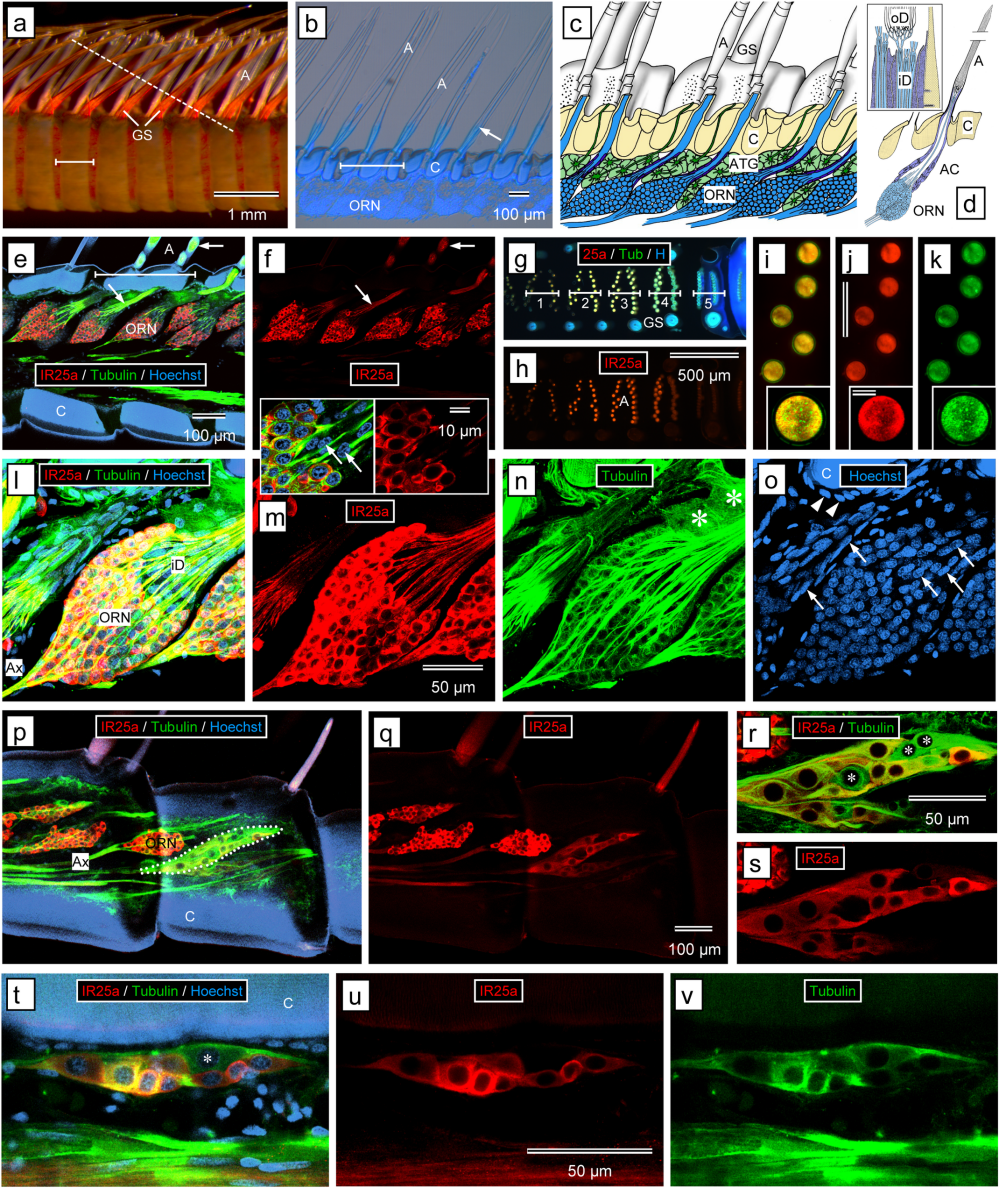
Immunolabeling with anti-HaIR25a in the lateral flagellum of the antennule. **(a–s)** Aesthetascs-bearing tuft region of the lateral flagellum. **(a)** Outer morphology—stereomicroscopical image. Each annulus (horizontal bar) bears two rows of transparent aesthetascs (A) accompanied by guard setae (GS). Dashed line shows direction of cross section through aesthetascs in **(g–k)**. **(b)** Sagittal section labeled with Hoechst 33258 showing full length of aesthetasc setae (A). Each annulus (horizontal bar) bears two rows of aesthetascs. ORN clusters (ORN) are labeled by Hoechst whereas cuticle (C) is autofluorescent. Only the proximal 20% of the cuticle of the aesthetasc setae containing the inner dendritic segments of the ORNs is autofluorescent (arrow). **(c)** Sagittal view of the lateral flagellum (modified from Schmidt et al. (2006) [87]). Each aesthetasc (A) is innervated by a cluster of ORNs (blue) whose inner dendritic segments traverse the cuticle (C) in a wide canal. ORN clusters are associated with subcuticular aesthetasc tegumental glands (ATG) whose thin drainage ducts terminate in pores at the base of the aesthetascs. Guard setae (GS) are located at the lateral margins of the aesthetasc rows. **(d)** Reconstruction of aesthetasc ultrastructure based on transmission electron microscopy; inset: region of transition from inner to outer dendritic segments within the base of aesthetasc setae at higher magnification (modified from Grünert and Ache (1988) [94]). Each aesthetasc (A) is innervated by about 320 ORNs whose somata form a cluster (ORN) below the cuticle (C). Inner dendritic segments arising apically from the ORN somata are wrapped by auxiliary cells (AC). Inset: each inner dendritic segment (iD) gives rise to two highly-branched outer dendritic segments (oD). **(e, f)** Sagittal section through medial plane of the tuft region of a lateral flagellum labeled with anti-HaIR25a (red), anti-tubulin (green), and Hoechst 33258 (blue) at low magnification (confocal images). Scale bar in **(e)** also applies to **(f)**. **(e)** Overlay of all three fluorescence channels. **(f)** anti-HaIR25a channel. Two rows of aesthetascs setae (A) arise from the intensely autofluorescent (blue) cuticle of an annulus (horizontal bar in **e**). Each aesthetasc seta is associated with a clearly delineated cluster of ORN somata (ORN). The somata and inner dendritic segments (arrows) are intensely labeled by anti-HaIR25a and anti-tubulin. **(g–k)** Cross sections through aesthetasc setae (direction of section indicated by dashed line in **(a)**). **(g, h)** Low magnification epifluorescence images; scale bar in **(h)** also applies to **(g)**. **(g)** Overlay of all three fluorescence channels. **(h)** anti-HaIR25a channel. Aesthetasc setae (A) located on different annuli (horizontal bars with numbers) are cut at different levels systematically progressing from the tips (1) to the base emerging from the annulus cuticle (5). Note that the lumen of the aesthetasc setae containing outer dendritic segments (1, 2, 3, 4—left aesthetasc row) is more intensely labeled by anti-HaIR25a than the lumen of aesthetasc setae containing only inner dendritic segments (4 –right aesthetasc row, 5). **(i–k)** High magnification epifluorescence images of sections through aesthetasc setae on annulus 3; insets: very high magnification (confocal images); scale bars in **(j)** (top: 50 μm; bottom: 10 μm) also apply to **(i)** and **(k)**. **(i)** Overlay of anti-HaIR25a and anti-tubulin fluorescence channels. **(j)** anti- HaIR25a channel. **(k)** anti-tubulin channel. Note that entire lumen of aesthetasc setae is filled by outer dendritic segments of ORNs intensely labeled by anti-HaIR25a and anti-tubulin. **(l–o)** Sagittal section through ORN clusters labeled with anti-HaIR25a (red), anti-tubulin (green), and Hoechst 33258 (blue) at high magnification (confocal images–maximum intensity projection of entire stack of optical sections spanning about 60 μm). Scale bar in **(m)** also applies to **(l)**, **(n)**, and **(o)**. **(l)** Overlay of all 3 fluorescence channels. **(m)** anti-HaIR25a channel. **(n)** anti-tubulin channel. **(o)** Hoechst channel. Insets in **(m)**: Apical region of ORN cluster at high magnification; left, overlay of all 3 fluorescence channels; right, anti-HaIR25a channel (confocal images of a single optical section). The somata (ORN), axons (Ax), and inner dendritic segments (iD) of all ORNs are intensely labeled by anti-HaIR25a and anti-tubulin. Somata of auxiliary cells (nuclei indicated by arrows) are not labeled by either of the antibodies and epithelial cells (nuclei indicated by arrowheads) and aesthetasc tegumental glands (asterisks) are labeled by anti-tubulin but not anti-HaIR25a. Autofluorescent (blue) cuticle (C). **(p–s)** Sagittal section through lateral plane of tuft region of lateral flagellum labeled with anti-HaIR25a (red), anti-tubulin (green), and Hoechst 33258 (blue). **(p, q)** Section at low magnification (confocal images); scale bar in **(q)** also applies to **(p)**. **(p)** Overlay of all three fluorescence channels. **(q)** anti-HaIR25a channel. In addition to clusters of ORN somata, clusters of sensory neurons innervating bimodal sensilla accompanying the aesthetascs (one cluster outlined by white dots) are also labeled by anti-HaIR25a and anti-tubulin. The intensity of labeling with anti-HaIR25a is higher in ORN somata and axons (Ax) than in somata and axons of the other sensory neurons. **(r, s)** Section at high magnification (confocal images; scale bar in **(r)** also applies to **(s)**. **(r)** anti-HaIR25a and anti-tubulin channel. **(s)** anti-HaIR25a channel. All sensory neurons of the clusters are labeled by anti-tubulin, but three of them (asterisks) mostly located in the distal aspect of the cluster are not double-labeled by anti-HaIR25a. **(t–v)** Sagittal section through cluster of sensory neurons innervating a bimodal sensillum in the proximal part of the lateral flagellum labeled with anti-HaIR25a (red), anti-tubulin (green) and Hoechst 33258 (blue). **(t)** Overlay of all three fluorescence channels. **(u)** anti-HaIR25a channel. **(v)** anti-tubulin channel. All sensory neurons of the clusters are labeled by anti-tubulin but one of them (asterisk) located in the distal aspect of the cluster is not double-labeled by anti-HaIR25a. The intensity of labeling with anti-HaIR25a and anti-tubulin differs substantially but independent of each other between labeled somata.

**Fig 6:**
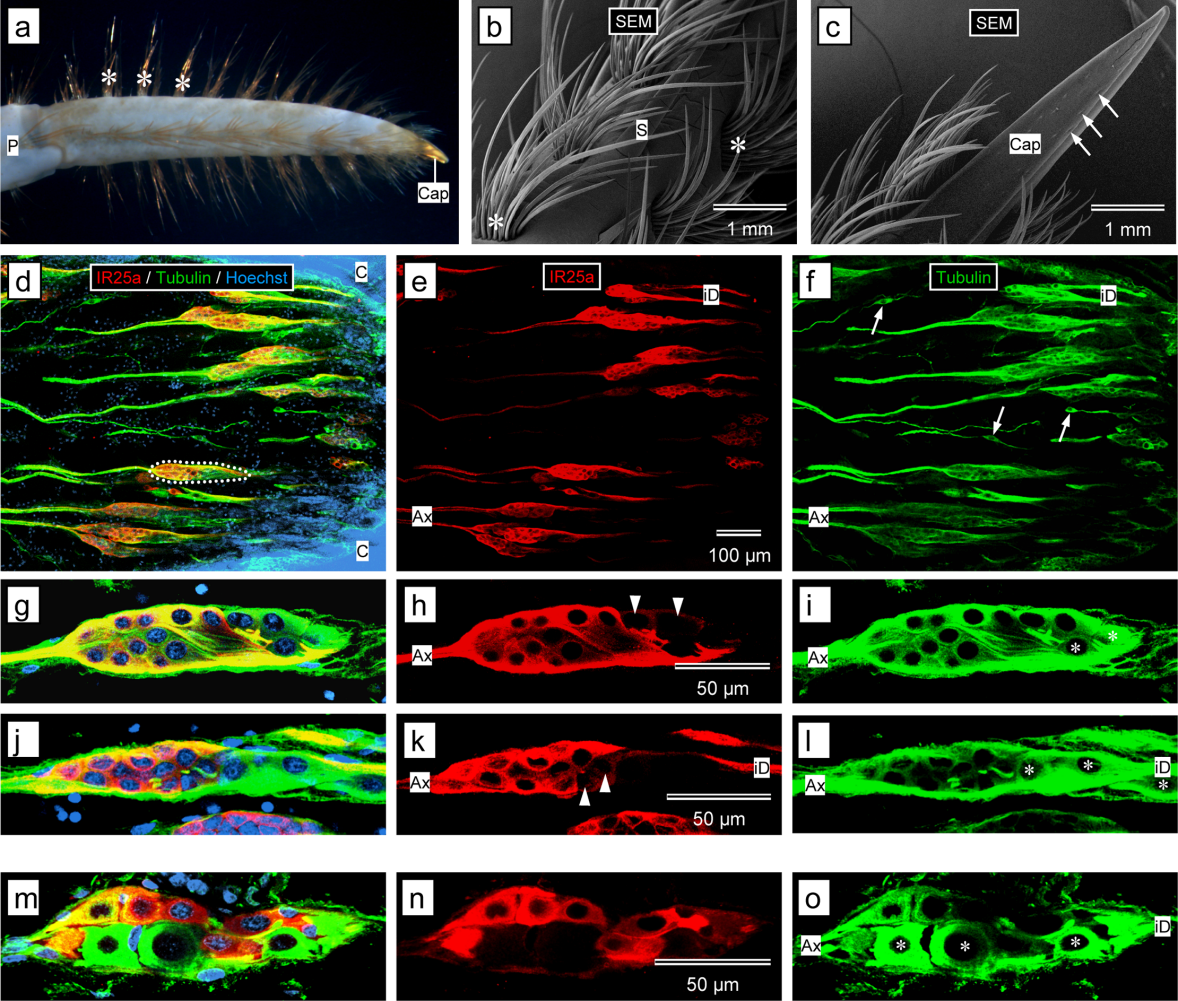
Immunolabeling with anti-HaIR25a in the walking leg dactyl and in the flagellum of the 2^nd^ antenna. **(a–l)** Walking leg dactyl. **(a)** Outer morphology of the dactyl of a third pereiopod of a late juvenile animal, shown in a stereomicroscopical image. The dactyl bears rows of evenly spaced bundles of smooth setae (asterisks), except on the epicuticular cap (Cap) at the tip. Propodus (P). **(b, c)** Outer morphology of the distal part of the dactyl of a second pereiopod of a late juvenile animal, shown in scanning electron micrographs (SEM). The main body of the dactyl bears rows of dense bundles of about 20 smooth setae (asterisks). Single smooth spines (S) are located between rows of bundled setae. The epicuticular cap (Cap) does not bear setae but instead holds numerous small depressions (arrows) that likely represent the outer structures of bimodal sensilla called funnel-canal organs. **(d–f)** Sagittal section through the distal aspect of a third pereiopod dactyl (proximal to epicuticular cap) labeled with anti-HaIR25a (red), anti-tubulin (green), and Hoechst 33258 (blue) at low magnification (confocal images); scale bar in **(e)** also applies to **(d)** and **(f)**. **(d)** Overlay of all three fluorescence channels. **(e)** anti-HaIR25a channel. **(f)** anti-tubulin channel. Numerous spindle-shaped clusters of sensory neurons (one outlined by white dots), each innervating one of the smooth bundled setae are intensely labeled by anti-HaIR25a and anti-tubulin. Both antibodies label the somata of sensory neurons as well as their axons (Ax) and inner dendritic segments (iD). Overlay of all three channels reveals that in the clusters of sensory neurons, neurons that are labeled by anti-tubulin but not by anti-HaIR25a (and therefore appear green) are located at the distal pole of the clusters. Single bipolar sensory neurons labeled by anti-tubulin but not anti-HaIR25a (arrows) are interspersed between clusters of sensory neurons. **(g–l)** Clusters of sensory neurons labeled with anti-HaIR25a (red), anti-tubulin (green), and Hoechst 33258 (blue) at high magnification (confocal image); scale bar in **(h)** also applies to **(g)**, **(i)**; scale bar in **(k)** also applies to **(j)** and **(l)**. **(g, j)** Overlay of all three fluorescence channels. **(h, k)** anti-HaIR25a channel. **(i, l)** anti-tubulin channel. Each cluster contains about 15 bipolar sensory neurons, all strongly labeled by anti-tubulin. Neurons in the proximal part of the cluster are also intensely labeled by anti-HaIR25a, but two or three neurons located at the distal pole of the cluster are not labeled by anti-HaIR25a and two other neurons in the distal region are only weakly labeled by anti-HaIR25a (arrowheads). Axons (Ax), inner dendritic segments (iD). **(m–o)** Flagellum of 2^nd^ antenna. Cluster of sensory neurons labeled with anti-HaIR25a (red), anti-tubulin (green), and Hoechst 33258 (blue) at high magnification (confocal images); scale bar in **(n)** also applies to **(m)** and **(o). (m)** Overlay of all three fluorescence channels. **(n)** anti-HaIR25a channel. **(o)** anti-tubulin channel. All sensory neurons of the cluster are labeled by anti-tubulin, although to different degrees. All but 3 sensory neurons are also labeled by anti-HaIR25a. Two of the HaIR25a-negative neurons (asterisks) are the largest neurons of the clusters suggesting that they are MRNs.

**Fig 7:**
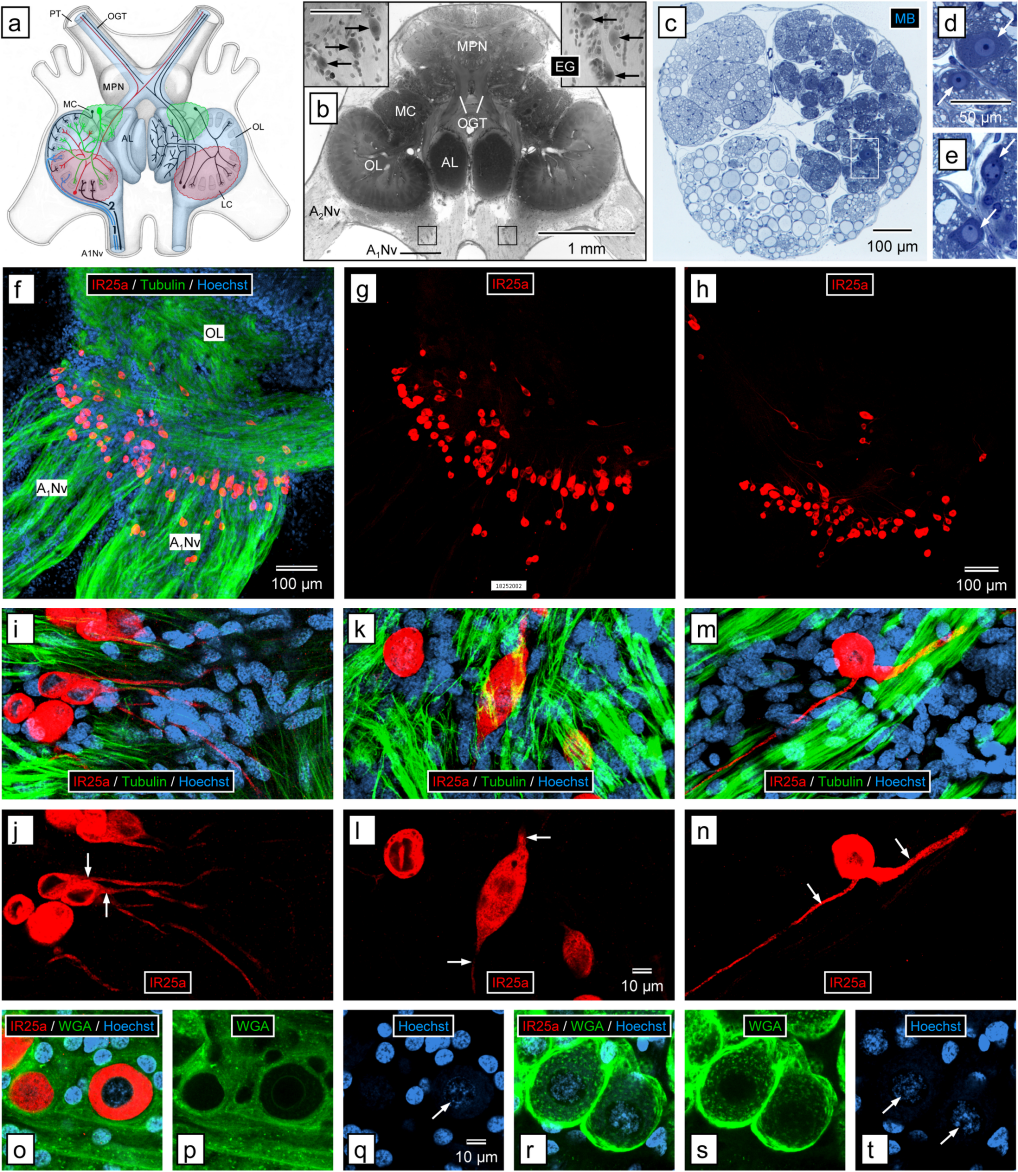
Immunolabeling with anti-HaIR25a in the brain. **(a)** Schematic drawing of the olfactory pathway (light blue overlay) in the brain of *P*. *argus* (modified from Schmidt and Ache (1996) [8]). Afferent axons of ORNs (1, blue) enter the brain via the antennular nerve (A_1_Nv) and project to the ipsilateral olfactory lobe (OL) where they terminate in one of its cone-shaped glomeruli. The OL is closely linked to another glomerular neuropil, the accessory lobe (AL). OL and AL are innervated by local interneurons (green) whose somata form the medial soma clusters (MC) and ascending projection neurons (red) whose somata form the lateral soma clusters (LC). Axons of projections neurons form the olfactory glomerular tracts (OGT) that run within the protocerebral tracts (PT) connecting the brain with the eyestalk ganglia. Median protocerebral neuropils (MPN). **(b)** Horizontal section through brain stained with ethyl gallate (EG). Large cells (arrows in insets) are located in the axon sorting zone of the A_1_Nv before it reaches the OL. Location of insets is shown by black squares; scale bar in left inset is 100 μm and also applies to right inset. Antenna 2 nerve (A_2_Nv). **(c–e)** Cross section through the antennular nerve where it enters the brain, stained with methylene blue (MB). **(c)** Low magnification. The axon fascicles in the antennular nerve form three large divisions. The lateral division is formed by axon fascicles from the lateral flagellum that are more intensely stained than other axon fascicles (because they contain numerous extremely thin ORN axons). Large, intensely stained cells selectively occur in the lateral division (white rectangle). **(d, e)** Large, intensely stained cells (arrows) in the lateral division of the antennular nerve at higher magnification. **(e)** Region highlighted in **(c)**. The large cells have voluminous cytosol and a spherical nucleus containing at least one dense nucleolus. **(f, g)** Confocal image of a sagittal section through brain labeled with anti-HaIR25a (red), anti-tubulin (green), and Hoechst 33258 (blue) at low magnification); scale bar in **(f)** also applies to **(g)**. **(f)** Overlay of all three fluorescence channels. **(g)** anti-HaIR25a channel. Anti-HaIR25a intensely labels a loose assembly of about 100 large cells located in the axon sorting zone of the A1Nv before it reaches the OL. Axons within the antennular nerve are intensely labeled by anti-tubulin but not by anti-HaIR25a, and HaIR25a-positive cells are not labeled by anti-tubulin. **(h)** Confocal image of a sagittal section through brain of different animal labeled with anti-HaIR25a at low magnification () shows a similar assembly of about 100 intensely labeled large cells in the axon sorting zone of the antennular nerve. **(i–n)** Morphology of single large cells in the axon sorting zone of the antennular nerve labeled with anti-HaIR25a (red), anti-tubulin (green), and Hoechst 33258 (blue) at high magnification (confocal images). Scale bar in **(l)** applies to all images. **(i, k, m)** Overlay of all three fluorescence channels. **(j, l, n)** anti-HaIR25a channel. **(i, j)** Unipolar cells. Most of the HaIR25a-positive cells have one process (arrows) projecting from the cell body. Generally, this process projects toward the OL. **(k, l)** Bipolar cell. Some of the HaIR25a-positive cells have two processes (arrows) projecting from both poles of the cell body. **(m, n)** Pseudo-unipolar cell. Rarely HaIR25a-positive cells have two processes (arrows) projecting from the same region of the cell body. **(o–t)** Double-labeling with anti-HaIR25a (red) and WGA-AF488 (Hoechst 33258—blue) at high magnification (confocal images). Scale bar in **(q)** applies to all images. **(o, r)** Overlay of all three fluorescence channels. **(p, s)** WGA-AF488 channel. **(q, t)** Hoechst 33258 channel. **(o–q)** Large cells in the axon sorting zone of the OL. **(r–t)** Neuronal somata in the medial soma cluster. Large cells in axon sorting zone are intensely labeled by anti-HaIR25a but not by WGA whereas somata in the MC are not labeled by anti-HaIR25a but are intensely labeled by WGA. Nuclei of large cells and neurons (arrows) are similar in shape (spherical) and in having very loose heterochromatin (Hoechst labeling of low intensity).

Given the importance of the co-receptor IR25a in forming functional receptors in sensory neurons in other arthropods, we sought to localize the expression of this protein in LF, dactyl, second antennae, and brain of *P*. *argus*. In all these tissues, anti-HaIR25a intensely and selectively labeled particular types of cells (Figs 5–7). Negative controls, by labeling tissue sections using the same ICC protocol but omitting anti-HaIR25a, showed no labeling of these cell types or any other cells. Use of anti-tubulin to label other tissue components and the nuclear marker Hoechst 33258 to reveal nuclei of all cells allowed a robust interpretation of the labeling pattern obtained by anti-HaIR25a. In LF, dactyl, and the flagellum of the second antennae (all of which contain no musculature), anti-tubulin primarily labeled bipolar sensory neurons, epithelial cells, and the walls of hemolymph vessels. Analysis of the overall distribution of cell nuclei strongly suggests that anti-tubulin indeed labeled all bipolar sensory neurons. In the brain, anti-tubulin primarily labeled neuronal elements, especially axonal tracts and neuropils.

**Lateral flagella of antennules**

In the aesthetasc-bearing tuft region of LF, anti-HaIR25a intensely labeled the clusters of ORN somata associated with the aesthetascs (Fig 5E–5Q) as shown previously [40]. Double-labeling with anti-tubulin revealed that most or all ORN somata of a cluster are HaIR25a-positive (Fig 5L–5O). Glia-like auxiliary cells of aesthetascs whose somata are located at the apical pole of ORN clusters surrounding the emerging inner dendritic segments (Fig 5D) were not labeled by anti-HaIR25a or anti-tubulin (Fig 5L–5O). In the ORN somata, HaIR25a-like immunoreactivity was not restricted to the cell membrane, but rather it extended throughout the entire cytosol. Labeling intensity within the cytosol was not uniform but more intense in spherical inclusions that may represent stacks of smooth endoplasmic reticulum (as described in ORN somata of another spiny lobster species [93]). The ORN somata in a cluster varied very little in size (n = 82, Feret diameter: 9.2–12.9 μm (minimum-maximum); mean ± SD: 11.0 ± 0.9 μm) and in the intensity of HaIR25a-like immunoreactivity. From the ORN somata, HaIR25a-like immunoreactivity and also tubulin-like immunoreactivity extended into the inner dendritic segments, which arise from the apical pole of the ORN somata, and into the axons, which arise from the basal pole of the ORN somata. For anti-HaIR25a, the labeling intensity was higher in the ORN somata than in the axons and inner dendritic segments (Fig 5M), whereas for anti-tubulin, the labeling intensity was higher in the axons and inner dendritic segments than in the somata (Fig 5N). Proximal to the ORN clusters, the axon fascicles arising from them fuse with the common lateral flagellum nerve which was intensely labeled by anti-tubulin and less intensely labeled by anti-HaIR25a (Fig 5P and 5Q). The inner dendritic segments arising from the apical pole of the ORN somata of a cluster form a common fascicle, which traverses the cuticle in a wide canal leading to the base of the associated aesthetasc seta (Fig 5C and 5D). Within the aesthetasc seta, at about 25% of the total setal length, the inner dendritic segments give rise to the outer dendritic segments representing modified cilia. The outer dendritic segments are highly branched and together the thin branches of all ORN outer dendritic segments fill the entire lumen of the aesthetasc seta distal to the transition area (Fig 5D). Cross sections through aesthetasc setae revealed that ORN outer dendritic segments were strongly labeled by anti-HaIR25a (Fig 5G–5J). In fact, labeling intensity was higher in the outer dendritic segments than in the inner dendritic segments or the ORN somata. Outer dendritic segments were also strongly labeled by anti-tubulin (Fig 5G, 5I and 5K), but in this case labeling intensity was lower in the outer dendritic segments compared to the inner dendritic segments.

In the tuft region of LF, aesthetascs are accompanied by three types of smooth sensilla likely innervated by distributed CRNs and MRNs [5, 95, 96]. These presumptive bimodal sensilla are guard setae, asymmetric setae, and companion setae (Figs 1D, 5A and 5C). They are located lateral to the two rows of aesthetascs traversing each annulus. Sections through the lateral region of the LF revealed the presence of clusters of bipolar sensory neurons that were distinctly labeled by anti-tubulin and anti-HaIR25a but clearly differed from clusters of ORNs in several parameters (Fig 5P–5S). First, the number of sensory neurons in a cluster was substantially lower (between 10 and 15) than in a typical cluster of ORNs (up to 320 [94]). Second, the overall intensity of HaIR25a-like immunoreactivity in these clusters was noticeably lower than in neighboring clusters of ORNs. Third, the somata of the sensory neurons composing these clusters were considerably larger on average than the somata of ORNs and they varied more in size (n = 45, Feret diameter: 8.8–30.1 μm (minimum-maximum); mean ± SD: 18.3 ± 4.8 μm. Fourth, not all sensory neurons delineated by distinct tubulin-like immunoreactivity were double labeled by anti-HaIR25a. Typically, two or three sensory neurons in the apical region of the cluster and occasionally single sensory neurons located further basally were completely devoid of HaIR25a-like immunoreactivity. Most likely each of these clusters of sensory neurons innervates one of the bimodal sensilla accompanying the aesthetascs, but we were unable to unequivocally link particular clusters to the setae they innervate. This was mainly because the inner dendritic segments arising apically from the somata were only weakly labeled by either antibody and therefore could not be traced to the base of setae. The axons arising basally from the somata were more intensely labeled by both antibodies (Fig 5P and 5Q). We failed to unequivocally detect anti-HaIR25a-like or tubulin-like immunoreactivity in the outer dendritic segments running through a narrow canal within the thick-walled setae. This was because the cuticle forming these setae is intensely autofluorescent in the channels used to visualize anti-HaIR25a-like or tubulin-like immunoreactivity.

Proximal to the aesthetasc-bearing tuft region, the LF of the antennule bears fewer and less regularly arranged presumptive bimodal sensilla of different types [95, 96]. Sections through the proximal region of the LF revealed the presence of widely dispersed clusters of bipolar sensory neurons that were distinctly labeled by anti-tubulin and anti-HaIR25a. These clusters of sensory neurons were similar to the ones presumably innervating bimodal non-aesthetasc sensilla in the tuft region of the lateral flagellum in number (between 8 and 15) and size (n = 8, Feret diameter: 10.4–20.4 μm (minimum-maximum); mean ± SD: 14.5 ± 3.4 μm) of sensory neurons and in the presence of one or two sensory neurons in the apical region of the cluster that were completely devoid of HaIR25a-like immunoreactivity. The inner dendritic segments arising apically from the somata were only weakly labeled by either antibody, and we were therefore unable to link the clusters to particular setae on the surface of the cuticle.

**Walking leg dactyl**

The dactyl of walking legs of *P*. *argus* is organized into a smooth epicuticular cap at the tip and a much longer proximal region that bears numerous dense bundles of robust smooth setae that are organized in six longitudinal rows (Fig 6A–6C). In addition, single smooth and very robust spines are interspersed between the rows of bundles of setae. In having a thin central canal that reaches all the way to their tip, the smooth setae and smooth spines show one morphological characteristic defining bimodal chemo- and mechanosensory sensilla [5]. The epicuticular tip bears numerous small depressions at the end of thin canals traversing the cuticle (Fig 6C). This organization is typical of bimodal sensilla called funnel-canal organs that lack an outer seta, and whose ultrastructure and chemosensory physiology was described in shore crabs, *Carcinus maenas* [91, 97, 98]. None of the presumptive sensilla on the dactyls of *P*. *argus* have been studied with electron microscopy, and chemoreception mediated by sensilla on the dactyls of *P*. *argus* has been demonstrated in only two studies [99, 100].

Sections through the distal region of the dactyl (excluding the epicuticular cap, which could not be sectioned well with the vibrating microtome even after decalcification) revealed the presence of numerous large clusters of bipolar sensory neurons that were distinctly labeled by anti-tubulin and anti-HaIR25a (Fig 6D–6F). These clusters of sensory neurons were similar to the clusters of sensory neurons that presumably innervate bimodal chemo- and mechanosensory sensilla in the LF (see above) in having a relatively low number of sensory neurons (between 15 and 20) that were larger than the somata of ORNs and varied substantially in size (n = 77, Feret diameter: 9.1–22.9 μm (minimum-maximum); mean ± SD: 13.9 ± 2.9 μm) (Fig 6G–6L). Most strikingly, also the clusters of sensory neurons in the dactyl typically had two or three sensory neurons (identified by their intense tubulin-like immunoreactivity) completely devoid of HaIR25a-like immunoreactivity in their apical region. In addition, the clusters contained one or two sensory neurons sub-apically that were only lightly labeled by anti-HaIR25a in contrast to the remaining neurons in the basal part of the clusters that expressed intense anti-HaIR25a-like immunoreactivity. Axon fascicles emerging basally from the clusters of sensory neurons and fascicles of inner dendritic segments emerging apically were intensely labeled by both antibodies. Fascicles of inner dendritic segments projected to the bases of bundled smooth setae but could not be traced further into the setae because of the intense autofluorescence of the setal cuticle. Interspersed between clusters of sensory neurons were single, spindle-shaped bipolar sensory neurons that expressed intense tubulin-like but no anti-HaIR25a-like immunoreactivity (Fig 6D and 6F).

**Second antennae**

The second antennae of *P*. *argus* are organized into three basal segments and an extraordinarily long and sturdy flagellum that is organized into numerous annuli. The anatomical organization of the annuli including their setation has not been studied in *P*. *argus* or any other spiny lobster species. Superficial examination of antennal flagella with light microscopy revealed that each annulus carries a stereotypical arrangement of setae concentrated at the distal edge of the annulus. Most of these setae are smooth and have a thin central canal that reaches all the way to their tip and hence show morphological characteristics of bimodal chemo- and mechanosensory sensilla [90]. Sections through the antennal flagellum revealed the presence of some clusters of bipolar sensory neurons that were distinctly labeled by anti-tubulin and anti-HaIR25a (Fig 6M–6O). These clusters of sensory neurons were located directly under the thick cuticle, and they were similar in number (between 6 and 12) and size of sensory neurons to the clusters of sensory neurons innervating bimodal non-aesthetasc sensilla in the distal and proximal lateral flagellum. One or two sensory neurons in the apical region of each cluster were completely devoid of HaIR25a-like immunoreactivity.

**Brain**

The neuroanatomy of the brain of *P*. *argus* is known in substantial detail especially with regard to the deutocerebrum that receives sensory input from sensilla on the antennules [7, 8, 92, 101, 102]. Axons of sensory neurons innervating sensilla on the antennules form the common antennular nerve that is subdivided into three main divisions. The lateral division contains axon fascicles originating from the LF including numerous extremely thin axons of ORNs, the medial division contains axon fascicles originating from the medial flagellum, and the dorsal division contains axon fascicles originating from the basal segments of the antennule [92]. Upon entering the brain, axons in the lateral division of the antennular nerve undergo a massive rearrangement in which the extremely thin ORNs axons get sorted out from all other axons in the lateral division. Proximal to this axon sorting zone, the ORN axons form a large fascicle projecting towards the olfactory lobe whereas all other axons form a fascicle projecting to the lateral lobe of the lateral antennular neuropil [92, 101]. The lateral division of the antennular nerve at its entry into the brain and the axon sorting zone contain conspicuous, very large cells (diameter 30–50 μm) that are intensely stained by ethyl gallate and methylene blue (Fig 7B, 7D and 7E; [101]) and were originally described by Herbst (1916) [103] in the antennular nerve of the spiny lobster, *Palinurus vulgaris*.

In the brain, anti-HaIR25a intensely and selectively labeled these large cells in the proximal part of the lateral division of the antennular nerve and the adjacent axon sorting zone (Fig 7F–7H). No other cellular structure in the brain including the ORN afferent axons that were intensely labeled by anti-tubulin (Fig 7F, 7I, 7K and 7M) expressed any anti-HaIR25a-like immunoreactivity. Labeling with anti-HaIR25a revealed that about 100 of the large cells are present in each hemibrain and that most of these cells (≥ 95%) are unipolar, giving off one process that typically projects towards the olfactory lobe (Fig 7I and 7J). A smaller fraction of large cells labeled by anti-HaIR25a (about 5%) were distinctly bipolar with a spindle-shaped soma and two processes arising from the opposing cell poles (Fig 7K and 7L). Very few large cells were pseudo-unipolar with two processes arising from one pole of the cell and projecting in opposite directions. Because the intensity of anti-HaIR25a declined rapidly in the processes, they could not be traced to their terminals. To elucidate if the large cells have neuronal identity, we double-labeled brains with anti-HaIR25a and WGA, which labels the vast majority of neurons residing in the CNS of *P*. *argus* [90]. In the large cells, WGA did not label the cytosol or the cell membrane, only the nuclear envelope was weakly WGA-positive. In contrast, neuronal somata in the same sections imaged using the identical setting of the confocal microscope showed intense labeling of the cytosol and very intense labeling of the cell membrane with WGA.

#### PCR

**Fig 8:**
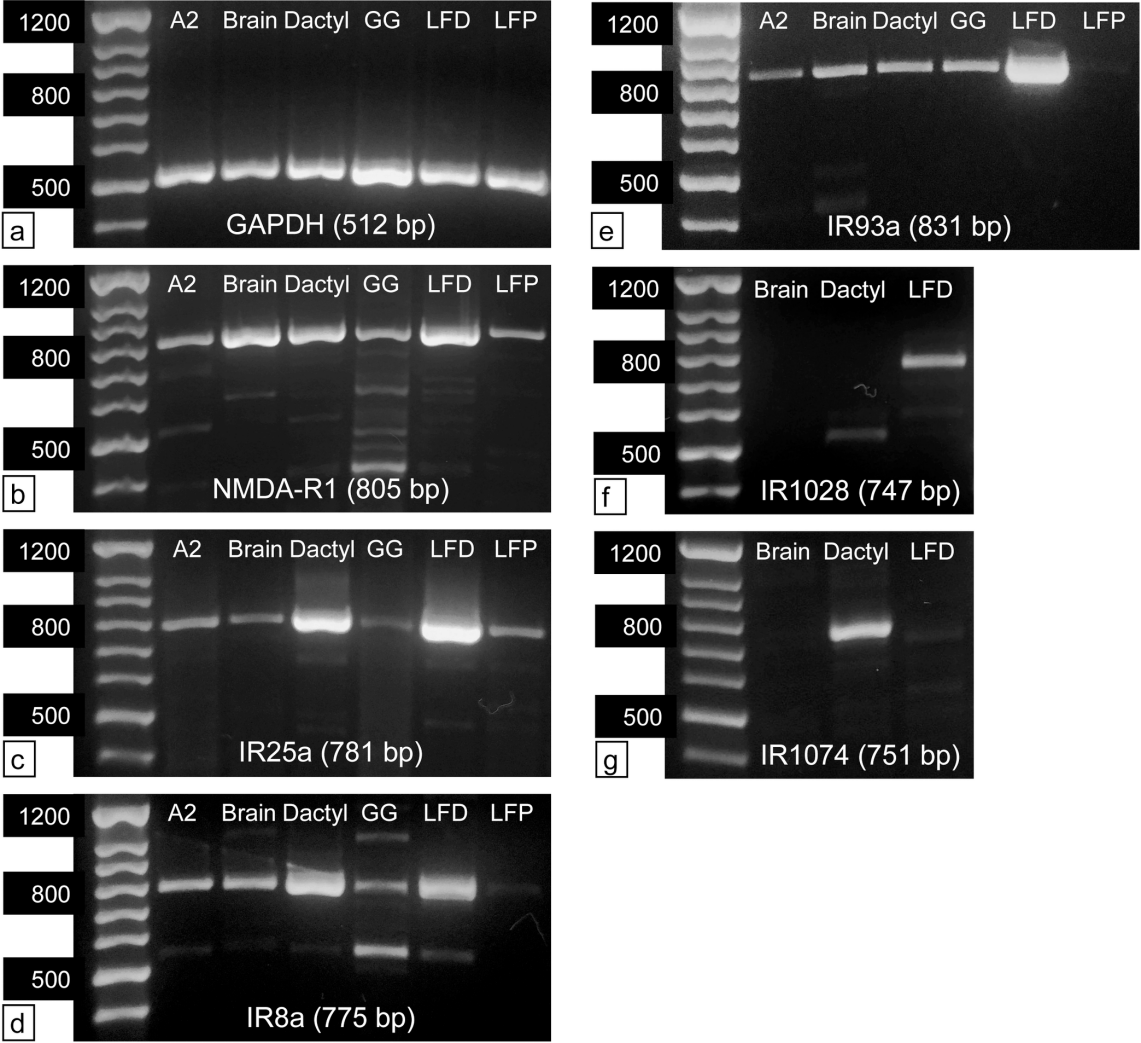
PCR results. **(a–e)** Gel images of PCR products amplified from all target tissues, antenna 2 (A2), central brain (Brain), dactyl of second pereiopod (Dactyl), green gland (GG), aesthetasc-bearing tuft region of the distal lateral flagellum of antennule (LFD), and proximal region of the lateral flagellum of antennule (LFP), using specific primer pairs for GAPDH **(a)**, NMDA-R1 **(b)**, IR25a **(c)**, IR8a **(d)**, IR93a **(e),** PargIR1028 **(f)** found only in LF; PargIR1074 **(g)** found only in dactyl. The predicted amplicon length of the PCR product for each primer pair is given in parentheses. The left side of each gel shows a 100 bp DNA ladder.

PCR was used to verify and extend the transcriptomics results by examining expression of IRs in an array of tissues that included those used to generate the Parg transcriptome but also extended to other tissues. We tested two regions of the LF, as described in the previous section: the distal region of the lateral flagellum of the antennule (LFD), which bears the olfactory (aesthetasc) sensilla; and the proximal, non-aesthetasc bearing region of the lateral flagellum of the antennule (LFP). Besides the dactyl of the pereiopods (Da) and the brain (Br), from which we developed transcriptomes, we also tested two other tissues: the second antenna (A2) and green gland (GG), an excretory organ. We examined three co-receptor IRs (IR25a, IR8a, IR93a) and two divergent IRs, the divergent PargIR1074 (expressed only in dactyl; S1 Fig and S2 Table) and the divergent PargIR1028, found in the Parg transcriptome. We also tested the iGluR, NMDAr1, and glyceraldehyde 3-phosphate dehydrogenase (GAPDH) as a positive control. Fig 8 shows representative results from our PCR runs, and Table 2 summarizes all findings. Our results show complete agreement between our transcriptomics and PCR results: genes expressed in a transcriptome were also found there through PCR. Thus, IR25a, IR8a, IR93a, and NMDAr1, identified in the Parg transcriptome, were also identified in all three tissues via PCR (Fig 8). IR1074, expressed only in dactyl and not LF or brain (S1 Fig and S2 Table), were also only found in dactyl via PCR. IR1028, expressed only in LF (S1 Fig and S2 Table), was also only found in LF via PCR (Fig 8). IRs expressed in the LF were expressed in both the LFD and LFP, although expression levels typically appeared to be qualitatively much higher in the LFD than LFP. PCR results showed that IR25a, IR8a, and IR93a are also found in the green gland, which is an excretory organ (Fig 8). We sequenced PCR products and verified that they have > 90% similarity to the expected sequence (Table 2).

**Table 2:** PCR results on expression of iGluRs and IRs in different tissues of *P*. *argus*.

Tissue	Controls		Co-receptor IRs			Tissue-specific IRs	
	GAPDH	NMDAr1	IR25a	IR8a	IR93a	IR1028	IR1074
**Dactyl**	**✓**	**✓**	**✓**	**✓**	**✓**	** **	** **
**Brain**	** **	**✓**	**✓**	**✓**	**✓**	** **	** **
**LFD**	**✓**	** **	**✓**	**✓**	**✓**	** **	** **
**LFP**	** **	**✓**	**✓**	** **	** **	**Not Tested**	
**A2**	** **	** **	**✓**	** **	** **		
**GG**	** **	** **	**✓**	**✓**	**✓**		

Green box indicates that a PCR product with the expected size was identified. A green box with a check mark indicates that the PCR product with expected size was identified and when sequenced was found to have > 90% similarity to the expected sequence. Blue box indicates that a PCR product of expected size was identified and was detected only in expected tissue as predicted from the transcriptomes. Blank box indicates the PCR product of expected size was absent in the tissue.

### Identification of a GR

**Fig 9:**
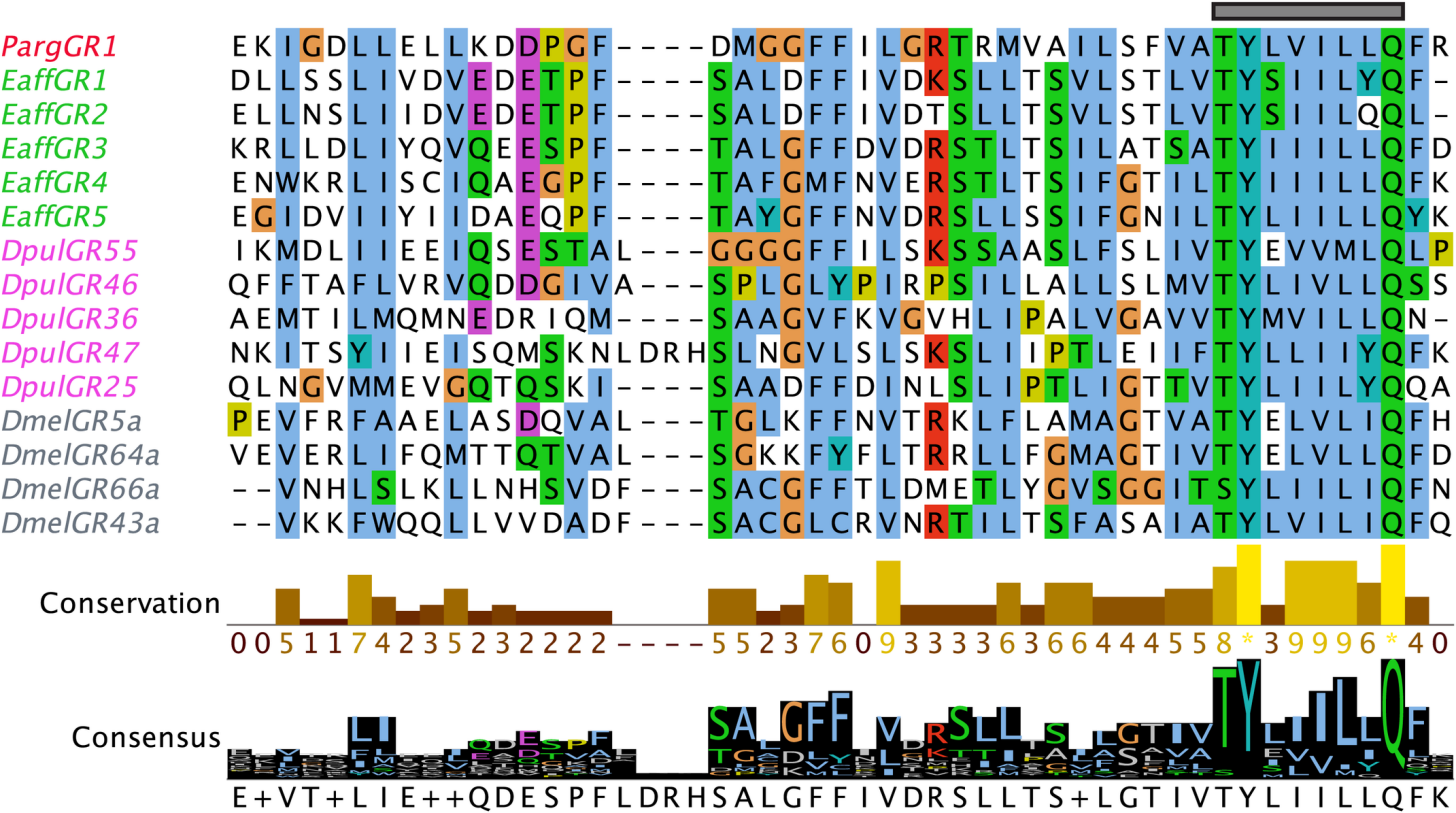
PargGR1 fragment sequence alignment. The multiple sequence alignment of PargGR1 (red) with GRs from arthropods, Eaff (green), Dpul (pink), and Dmel (grey) shows the TM7 region of the 7tm_7 superfamily. Sequences were aligned using MAFFT and visualized on Jalview. Conservation of amino acids across GRs of various species is highest at the ‘TYxxxxxQF’ motif (grey bar) as shown in the consensus histogram. The residues were colored according to the Clustal X color scheme on Jalview.

We searched for insect-like ORs and GRs in the Parg transcriptomes. We did not find any ORs, but we did find one GR, PargGR1 (Fig 9, S11 and S12 Texts), according to the following lines of evidence as suggested by Robertson (2015) [14]. First, BLAST searches of our Parg transcriptomes using arthropod GRs as query files identified PargGR1 as a GR (see **Methods** for e-values). Second, the top hits from BLAST searches of non-redundant NCBI databases with PargGR1 as the query were arthropod GRs. Third, an InterProScan screen of our Parg database for the GR specific 7tm_7 domain, PF08395, identified PargGR1 [14, 104]. The screen using InterProScan did not produce other candidates with OR specific 7tm_6 domain, PF02949, or the 7tm_7 domain in the Parg transcriptome. Although the PargGR1 sequence is a fragment (134 amino acids), Fig 9 shows the conserved region of the 7tm_7 domain [14] for PargGR1 along with several GRs from Dmel, Dpul, and the copepod *Eurytemora affinis* (Eaff) acquired from Eyun et al. (2017) [31], demonstrating the presence of 7tm_7 in the PargGR1 fragment. Consistent with GRs and GRLs (GR-Like) from other species, PargGR1 also has the highly conserved motif ‘TYxxxxxQF’ found in the TM7 region of all GRs [14, 51]. Using TMHMM, we were only able to predict one TM region in PargGR1 due to the small size of the fragment.

### Identification of TRP channels

We searched our Parg protein database for Transient Receptor Potential (TRP) channels by using BLASTp and TRP channel sequences as queries from insects (Dmel, Bmor, Tcas, Amel, Nvit, and Phum), nematodes (Cele), and mammals (Mmus and Rnor). Using the BLAST results and an InterProScan screen, we selected Parg sequences with TRP channel domain regions (see **Methods** for list of Pfam domains) from the transcriptome, and then used these sequences along with the TRP channel query sequences and Dpul TRP channels [63] to construct a phylogenetic tree.

**Fig 10:**
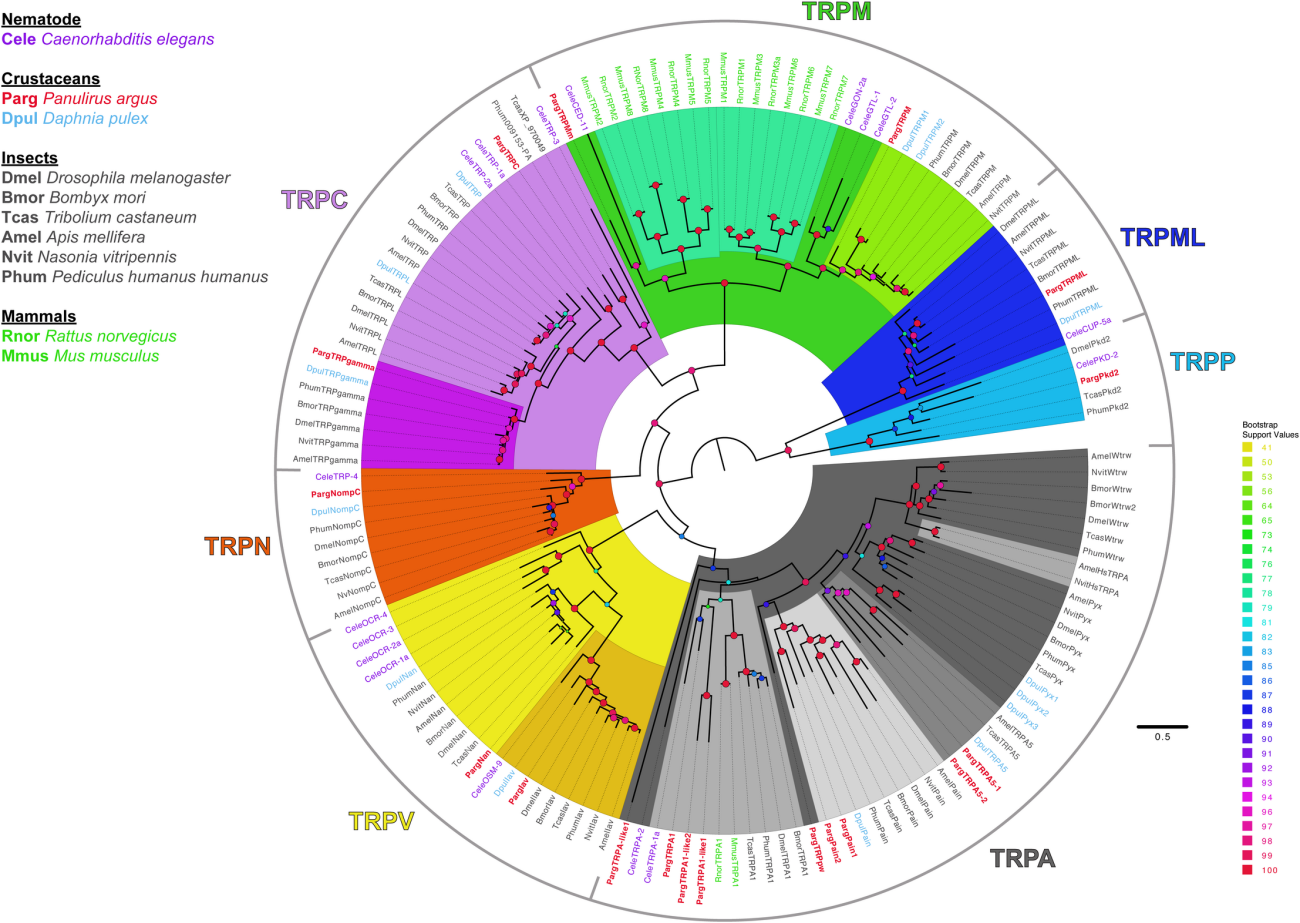
Maximum likelihood phylogenetic tree of TRP channels. Various subfamilies of TRP channels are indicated by different colors where shades of each color indicates a class of TRP channels within a subfamily: TRPA subfamily (grey), TRPM (green), TRPV (yellow), TRPC (pink), TRPN (orange), TRPP (light blue), and TRPML (dark blue). Sequences were aligned with MAFFT and visualized on Jalview. The tree was built on IQ-Tree under LG+G4 model of substitution with 1000 UFBoot replications and visualized on FigTree v1.4.2. Tree was unrooted but drawn with Pkd2 and TRPML clades as the root. The scale bar represents expected number of substitutions per site.

Homologues to all seven TRP subfamilies [56, 57] were found in the Parg transcriptome (Fig 10; S13–S15 Texts). An expanded TRPA subfamily of the Group 1 TRP channels of insects was identified that was similar to insects [85]. Overall, our analysis determined nine types of TRPA channels in the Parg transcriptome. One homologue to TRPA1 was identified and found to cluster with Cele TRPA1. Two more homologues to TRPA1, PargTRPA1-like1 and PargTRPA1-like2, were also detected. Two homologues to painless, an insect TRPA channel, were identified. Two homologues to hymenopteran TRPA5 channels, PargTRPA5-1 and PargTRPA5-2, were also detected in the transcriptome (S13–S15 Texts). Another TRPA subfamily member, PargTRPApw, clustered with the insect TRPA channel clades pyrexia, waterwitch, and painless. The remaining member of the TRPA subfamily, PargTRPA-like1, clustered with the TRPA subfamily clade but did not directly cluster with any of the TRPA channel types in insects or Dpul.

Two homologues to the TRPM subfamily were identified. The first, Parg TRPM, is a homologue to the insect TRPM subfamily [85] (Fig 10; S13–S15 Texts). The second, Parg TRPMm, is a homologue to the mammalian TRPM channel subfamily and clustered with the Cele TRP channel, CED-11. PargTRPMm had higher abundance estimated in the LF (S2 Fig and S3 Table).

Two homologues to the TRPV subfamily were found. A homologue to the arthropod TRPV channel ‘Nanchung,’ PargNan, and the homologue, PargIav, to the TRPV channel ‘inactive’ in arthropods and OSM-9 in Cele were found in the Parg transcriptome. Two homologues to the classical TRP channels, TRPC subfamily, were identified. The first, PargTRPgamma, is homologous to arthropod TRPgamma channels. The second, PargTRPC (Fig 10; S13–S15 Texts) clusters with the Cele TRP-1a channel. A single homologue, PargNompC, to the insect TRPN channel, NompC, was identified.

Homologues to the group 2 TRP subfamilies, TRPP (Pkd2) and TRPML, were also found in Parg (Fig 10). A single homologue, Parg TRPML, to insect TRPML sequences and the homologue to insect Pkd2 channel, PargPkd2, were found.

## Discussion

The overarching aim of this study is to gain a molecular understanding of the first step in chemoreception–the receptor proteins on olfactory and distributed chemoreceptor neurons–in the Caribbean spiny lobster, *Panulirus argus*, from a functional and phylogenetic perspective. We chose *P*. *argus* because it is a major model decapod crustacean for chemoreception and thus represents a good starting point for comparison with other decapod crustaceans and other arthropods, in particular insects. Our principal objective was to identify and compare a major set of chemoreceptor proteins of insects–the ionotropic receptors (IRs)–in the two major chemosensory organs–the antennular lateral flagellum representing olfaction and distributed chemoreception, and the leg dactyls representing only distributed chemoreception–and brain of *P*. *argus*. A secondary objective was to search for other types of chemoreceptor proteins besides IRs, including Olfactory Receptors (ORs), Gustatory Receptors (GRs), and Transient Receptor Potential (TRP) channels. We conservatively found 108 IRs that include co-receptor IRs, conserved IRs, and divergent IRs. However, most (95) are divergent IRs and the vast majority (51) of these divergent IRs are found only in the antennular lateral flagellum and thus likely involved in olfaction. We show that one co-receptor, IR25a, is expressed in olfactory receptor neurons and other chemosensory neurons. We also identified one GR and homologues to all subfamilies of TRP channels. Some of these molecules are expressed not only in chemosensory tissue but also in the brain, suggesting that they may mediate diverse functions.

### Diversity and distribution of IRs in two chemosensory organs of *P*. *argus*

We identified 108 IRs in the antennules, leg dactyls, and brain of *P*. *argus*: four co-receptor IRs, nine conserved IRs, and 95 divergent IRs. This is a conservative estimate of the diversity of IRs, because we only included sequences with both the ligand binding domain and the ion channel domain that did not have large gaps in these domain regions.

The four IR co-receptors of *P*. *argus*–IR8a, IR25a, IR76b, and IR93a –were found in both the LF and dactyl of *P*. *argus*. IR25a has been found in all protostomes examined to date with few exceptions, suggesting it is the ancestral IR and has conserved functions [25, 31]. IR8a, IR76b, and IR93a evolved more recently, and their expression appears to be limited to the arthropods. IR25a and IR8a have been found in most crustaceans examined to date, including *Homarus americanus* [39], spiny lobsters [40, 41], seven species of shrimp [44], and copepods [31, 45–47]. Some crustaceans have been found to have IR25a but not IR8a, including the branchiopod *Daphnia* and two species of hermit crabs (Fig 4) [25, 42, 43]. Eyun et al. (2017) [31] propose that IR8a appeared early in the pancrustaceans and may have been secondarily lost in the branchiopods. IR76b has been found not only here in *P*. *argus* but also in copepods [31] and *Daphnia*. The relatively infrequent reporting of IR76b in crustaceans might be because it has only recently been identified in *Daphnia* ([31], Fig 4) and could not be identified in phylogenetic analyses without homologues from other crustaceans.

Orthologues of some IRs have been found across insects. These are called conserved IRs and include IR21a, IR31a, IR40a, IR41a, IR60a, IR64b, IR68a, the IR75 family, IR76a, IR84a, and IR92a (Fig 4) [25, 27]. Some of these insect conserved IRs–IR21a and IR40a –have also been found in other arthropods, including some chelicerates, myriapods, and crustaceans [31]. We also identified several homologues of IR40a and IR75-like, one homologue of IR21a, and one putative homologue of IR68a in *P*. *argus*. Eyun et al. (2017) [31] did not find IR40a in the 14 species of examined crustaceans.

The functions of IRs have been most explored in insects, especially *D*. *melanogaster*. In crustaceans, IR25a appears to be broadly expressed in all chemosensory neurons, but this is not the case in *D*. *melanogaster*. Although IR25a is broadly expressed in a subset of *D*. *melanogaster* antennal sensilla (coeloconic sensilla), it is not expressed in all ORNs in those sensilla. IR8a is also a co-receptor IR in *D*. *melanogaster* coeloconic sensilla. However, IR25a and IR8a are not expressed in the same neurons, with a few exceptions [24, 26]. They form functional ionotropic receptors that respond to specific chemicals when co-expressed with specific divergent IRs [26, 27, 32]. These co-receptor IRs can play an integral role in the transport and insertion of IRs into the sensory ciliary membrane [26]. IR25a of *D*. *melanogaster* is a co-receptor not only in chemosensory neurons but also in neurons sensing humidity and cool temperatures [105–107]. IR25a has also been implicated in mediating the circadian clock by itself through warmth-sensitivity without the co-expression of other IRs [108].

Another co-receptor IR found across arthropods, IR93a, is expressed in antennal neurons in *D*. *melanogaster*, and it functions in hygroreception together with IR25a and the conserved IRs IR40a and IR68a [105, 106, 109, 110]. Another conserved IR, IR21a, plays a role in thermoreception when co-expressed with IR25a and IR93a [106, 107]. We do not know the role of these conserved IRs in *P*. *argus* and other crustaceans. We detected a putative homologue to IR68a in *P*. *argus*, and found several homologues to IR40a and one to IR21a (Figs 2 and 4), raising the possibility that in crustaceans IR25a and IR93a might play a role in reception of stimuli beyond chemicals. Previously, IR93a was found only in antenna of insects, but we found this co-receptor IR not only in the LF but also in the dactyl and brain of *P*. *argus*. The expansion of IR40a homologues and the possible presence of IR68a in *P*. *argus* is particularly interesting since IR40a mediates ‘dry’ humidity sensing and IR68a mediates ‘moist’ humidity sensing in *D*. *melanogaster* when co-expressed with IR25a and IR93a [105, 106, 109, 110].

The co-receptor IR76b is broadly expressed in the antennal ORNs of *D*. *melanogaster* but is typically co-expressed with IR25a or IR8a. The co-expression of the conserved IR, IR41a, in IR76b expressing ORNs confers them with polyamine sensitivity [34]. IR25a and IR76b are not only expressed in the antenna of *D*. *melanogaster* but are also broadly expressed in the gustatory receptor neurons (GRNs) in sensilla on the labellum and tarsi [25, 111, 112]. Only IR76b appears to function as a co-receptor in these GRNs despite co-expression with IR25a. IR76b-expressing GRNs in the labellum and tarsi detect amino acids [33, 112], salt [111], and polyamines [34]. Furthermore, salt detection in tarsal GRNs is blocked when the divergent IR, IR20a, is co-expressed with IR76b [33, 112]. Given this, it is interesting to speculate that in CRNs of crustaceans, the divergent IRs co-expressed with IR76b might also confer a particular chemical sensitivity to that neuron.

In insects, IRs are preferentially sensitive to amines and acids, whereas ORs are more sensitive to esters and alcohols [26, 32]. This bias toward amines and acids for insect IRs is consistent with the evolution of IRs in ancestral protostomes from iGluRs, which are sensitive to L–glutamate. Abuin et al. (2011) [26] proposed that IR25a, which is most similar in sequence to iGluRs and is likely the ancestral IR, functioned as a glutamate detector in common protostome ancestors that lived in marine environments. With the expansion of IRs, IR25a and IR8a assumed co-receptor functions and the new divergent IRs allowed sensitivity to a broader array of environmental chemicals. What might be the ligands for the IRs of crustaceans? We know that for *P*. *argus* and other crustaceans, many olfactory and distributed chemoreceptors neurons are sensitive to amino acids, amines, and other small nitrogenous molecules [5, 6, 113], which are of the same general classes though different specific molecules than detected by IRs of insects. Assuming that IRs are the major if not sole chemoreceptor proteins on ORNs and CRNs, then amino acids, amines, and other small nitrogenous molecules are among the ligands for IRs. Of course, identifying the specific ligands for each divergent IR will require future studies.

A total of 95 divergent IRs were identified in the LF and dactyls of *P*. *argus*. This is a conservative estimate, and the number of divergent IRs may be as high as 300. We found several unique sequences in the Parg transcriptome that had only one domain region (PF00060: transmembrane domains, pore, and S2 of LBD). All these sequences were not used for phylogenetic analyses and instead only a small subset that also had the S1 domain region were analyzed. Since we required that identified IRs have both domain regions (i.e., PF00060 and PF10613), our phylogenetic analyses did not include all sequences that had only one of the two domains regions. Interestingly, these 95 divergent IRs have different expression patterns in LF and dactyl: 51 are expressed only in LF, two are expressed only in the dactyl, 14 are expressed in both LF and dactyl, and the rest are expressed in LF and brain. One possible reason for this difference is that LF contains both olfactory and distributed chemoreceptor sensilla and neurons, while the dactyl contains only distributed chemoreceptors. If the number of IRs associated with distributed chemoreception were the same in LF and dactyl, this would imply that most IRs in the LF are part of the olfactory pathway. We know that the olfactory pathway of *P*. *argus* mediates responses not only to food-related chemicals (as do dactyl chemoreceptors) but it also uniquely mediates responses to conspecific chemicals such as social cues, alarm cues, and most probably sex pheromones[5, 6, 9]. Despite the fact that both the LF and dactyl have sensilla that are innervated by distributed chemoreceptors, the overlap of IRs between these two organs is low. This finding suggests that all populations of distributed chemoreceptors on other appendages (Fig 1) express distinct sets of IRs, which makes the overall chemosensory system in *P*. *argus* unexpectedly complex. Alternative explanations are possible, of course, one of which is that distributed chemoreceptors express another class of currently unidentified chemoreceptor proteins not expressed in ORNs and that these unidentified proteins will significantly raise the total number of protein types expressed in CRNs to be equal to that of the IRs in ORNs.

How does the total number of IRs in *P*. *argus* compare to other species? By our conservative estimate, the number is about the same in insects: *Drosophila* spp. have 58–69 IRs, and other insect species are often in this range though the number in insects can be as few as 20 or as many as 150 [27]. Insects also have many ORs and GRs. Among crustaceans, *Daphnia* has 85 IRs and 58 GRs and thus seems to be highly equipped with chemoreceptor proteins despite its highly reduced olfactory system (both antennule and brain) relative to the complex lifestyle and use of chemical sensing in *P*. *argus*. The number of divergent IRs in hermit crabs has been reported to be lower: 16 in the hermit crab *Pagurus bernhardus* and 22 identified in *C*. *clypeatus* [42].

#### Cellular expression patterns and possible functions of crustacean IRs

**IR25a in the periphery**

In all sensilla-bearing appendages included in this study (lateral flagella of antennules, leg dactyls, second antennae), anti-HaIR25a exclusively labeled sensory neurons and no other tissue components. This high degree of selectivity is in line with the notion that the antibody indeed binds to authentic IR25a as was demonstrated for *P*. *argus* IR25a with Western blots [41] and for *H*. *americanus* IR25a through preabsorption controls with the antigen and Western blots [48]. Immunocytochemistry results for *P*. *argus* (Figs 5 and 6; [40, 41]) suggest that IR25a is expressed in most or all ORNs in the antennules and all CRNs but not MRNs in antennules, legs, and second antennae.

Intense labeling with anti-HaIR25a was previously reported for the clusters of ORN somata associated with the aesthetascs in *H*. *americanus*, *P*. *argus*, and the land hermit crab *Coenobita clypeatus*. This broad expression across the ORNs and CRNs in crustaceans is suggestive of a co-receptor function. In addition, a matching labeling pattern was found in *H*. *americanus*, *P*. *argus*, and *C*. *clypeatus* with *in situ* hybridization using specific antisense probes for IR25a [40, 41, 43, 48]. Our results corroborate the previous findings in *P*. *argus*. Furthermore, double labeling with anti-tubulin antibody, which appears to label all sensory neurons (albeit not selectively), demonstrates that in fact all ORN somata of a cluster are IR25a-positive, which also appears to be the case in *H*. *americanus* and *C*. *clypeatus*. The apparent ubiquitous and uniform (low variation in labeling intensity) expression of IR25a in all ORNs in decapod crustaceans contrasts sharply with the expression pattern of chemoreceptor proteins in ORNs of *D*. *melanogaster* and other insects. First, olfactory sensilla of insects are differentiated into distinct morphological classes (and can occur on mouthpart appendages in addition to the antenna), and only one class (coeloconic sensilla) is innervated by IR-expressing ORNs whereas the majority of sensilla (trichoid and basiconic sensilla) are innervated by ORNs expressing ORs and/or GRs [24, 32]. Furthermore, in *D*. *melanogaster*, IR-expressing ORNs are differentiated into some in which IR25a is expressed highly (intensely labeled by anti-DmIR25a - some additional ORNs are weakly labeled) and acts as co-receptor, and others in which IR8a is highly expressed (intensely labeled by anti-DmIR8a) and acts as co-receptor [26]. The different pattern suggests an evolutionary trajectory leading from a single class of ORNs in crustaceans characterized by expressing IRs, having IR25a as a co-receptor, and innervating a morphologically uniform sensillum type (aesthetascs), to multiple classes of ORNs in insects expressing chemoreceptor proteins of different types (IRs, ORs, GRs), having IR-expressing ORNs with different co-receptors (IR25a or IR8a), and innervating sensilla of distinct morphologies.

Clues about the possible functions of IR25a in the ORNs of *P*. *argus* are provided by the distribution of IR25a-like immunoreactivity in different cellular compartments. Of all labeled cellular compartments (outer dendritic segments, inner dendritic segments, somata, initial axon segments), the outer dendritic segments showed the highest intensity of anti-HaIR25a-like immunoreactivity, suggesting that they are the primary site of IR25a function. This finding corroborates a previous report that described IR25a expression in the outer dendritic segments of *P*. *argus* ORNs [41] and is in line with the distribution of anti-DmIR25a-like immunoreactivity in *Drosophila* ORNs [24, 26]. Since the outer dendritic segments are the sites of odorant binding and sensory transduction [114, 115], identifying IR25a protein in this cellular compartment strongly suggests that it is part of the receptor complex (and hence directly contributes to sensory transduction) and/or it is involved in the trafficking of other IRs (divergent IRs) to the dendritic membrane as has been demonstrated for IR25a expressed in *Drosophila* ORNs [26]. If the outer dendritic segments are the primary location of IR25a in the ORNs, the weaker labeling of the somata and inner dendritic segments with anti-HaIR25a suggests that IR25a is synthesized in the somata and then transported through the inner dendritic segments to its final target. Labeling of the initial axon segments with anti-HaIR25a and the complete lack of labeling of the ORN axon terminals in the OLs of the brain matches the labeling pattern of *D*. *melanogaster* ORNs with anti-DmIR25a and anti-DmIR8a [24, 26]. In contrast, ORN axon terminals are intensely labeled by anti-HaIR25a in *H*. *americanus* [116], suggesting substantial interspecies differences in the function of IR25a in ORN axons of decapod crustaceans. In ORNs of mammals, ORs are expressed throughout the axons including their terminals [117, 118], and these axonal ORs contribute to axon targeting into select glomeruli [119]. In insect ORNs, expression of ORs was detected in axons, but ORN axon targeting is OR-independent [120] and it has not been determined if OR expression extends into the axon terminals in the antennal lobe [121, 122].

In addition to labeling ORNs, anti-HaIR25a intensely labeled other sensory neurons in all appendages or their parts (distal and proximal part of the lateral flagellum) included in this study–a finding that has not been reported previously. In each case, 6–24 HaIR25a-posititve sensory neurons were located in spindle-shaped clusters of sensory neurons (delineated by anti-tubulin labeling) that contained 1–5 additional neurons at their apical pole that were not or only very weakly labeled by anti-HaIR25a. In number and location, these clusters of sensory neurons closely correspond to populations of diverse types of setae with morphological characteristics of bimodal chemo- and mechanosensory sensilla, but we could not unequivocally establish the connection of clusters of sensory neurons to particular setae, because of the long distance and weak labeling of the other dendritic segments. However, since we did not find any clusters of sensory neurons labeled by anti-tubulin that did not contain HaIR25a-positive neurons, we conclude that likely all bimodal chemo- and mechanosensory sensilla in the tested appendages of *P*. *argus* are innervated by a group of sensory neurons expressing IR25a and a few others that do not. Preliminary results from labeling other appendages (third maxillipeds, uropods, pleopods) with anti-HaIR25a strongly indicate that this finding can be extrapolated to the entire distributed chemoreception system of *P*. *argus* (M. Schmidt, S. Sparks, and C. Derby, unpublished data). Comparing the numbers of HaIR25a-positive and HaIR25a-negative sensory neurons in a cluster with the numbers of presumptive CRNs and MRNs innervating diverse bimodal chemo- and mechanosensory sensilla on the antennules (8–10 / 12–13 / 15 total number of sensory neurons, 3–4 of which are MRNs [95, 96]) and mouthpart appendages (11–17 / 33–41, 1–4 of which are MRNs [123]) of *P*. *argus* indicates that anti-HaIR25a selectively labels CRNs while MRNs are HaIR25a-negative. All presumptive CRNs in the clusters of sensory neurons innervating bimodal sensilla on the second antenna and both parts of the LF are distinctly HaIR25a-positive (in parallel to the situation in ORNs). However, this is not the case in the clusters of sensory neurons innervating dactyl sensilla. Here, 2–4 of the presumptive CRNs are only very weakly labeled by anti-HaIR25a, suggesting that they may express a different IR as primary co-receptor. In a subset of *D*. *melanogaster* IR-expressing ORNs, IR8a serves as co-receptor, and given the particularly intense bands of IR8a in the dactyl (Fig 8D), which though non-quantitative is consistent with high expression, allows for the possibility that IR8a may also be a co-receptor in dactyl sensilla.

Only in the dactyl did we identify single tubulin-positive bipolar sensory neurons that were HaIR25a-negative, suggesting that they represent MRNs innervating unimodal mechanosensory sensilla. In the tip region of dactyls of shore crabs, *Carcinus maenas*, unimodal mechanosensory sensilla innervated by two sensory neurons (called intracuticular sensilla) are known [124], and it is possible that *P*. *argus* dactyls bear similar sensilla.

Our PCR results confirmed the expression of IR25a and two other co-receptors (IR8a, IR93a) in tissues containing ORNs and/or CRNs, including the proximal and distal regions of the LF, walking leg dactyls, and second antennae. PCR also confirmed the tissue specificity of one divergent IR expressed only in the LF (IR1028) and one IR expressed only in the dactyl (IR1074). Zbinden et al. (2017) [44] used PCR to identify IR25a expression in LF and medial flagellum and second antennae of several species of shrimp, but contrary to our results in *P*. *argus*, they did not identify it in the walking legs or mouthparts of shrimp.

IR25a in the CNS

In the brain, anti-HaIR25a exclusively and very distinctly labeled large cells in the lateral division of the antennular nerve (containing the axons of ORNs) and the axon sorting zone proximal to the OL. The axons and axon terminals of the ORNs, however, were completely devoid of labeling. This is unexpected, because the axons are clearly labeled further distally (at their origin from the ORN somata; see above) and because in *H*. *americanus*, the axon terminals of ORNs in the OL are distinctly HaIR25a-positive [116]. The distinct labeling by anti-HaIR25a allowed further characterization of the hitherto unknown morphological properties of these large cells. All cells possess at least one long, thin process, clearly identifying them as either neurons or glial cells [90]. The processes project in the direction of the OL, but because the labeling fades out rapidly (typically in less than 100 μm from the cell soma), the target of the processes and their terminal structures remain unknown. It is possible that thick fibers with multiglomerular arborizations in the outer fibrous layer of the olfactory lobe labeled by backfilling the antennular nerve that were interpreted as terminals of MRNs [101] are in fact the projections of the large cells. Filling the large cells with an intracellular marker would be required to substantiate this speculation.

Even the seemingly simple question if the large cells are neurons or some type of glial cells has no straightforward answer at present. In his original description of these cells in the antennular nerve of the spiny lobster, *Palinurus vulgaris*, Herbst (1916) [103] stated that they are neurons (‘Ganglienzellen’) based on being of similar size as neurons of the brain. However, since both neurons and glial cells of the spiny lobster brain are diverse in size [90], this argument is not conclusive. Our data provide additional evidence for a neuronal identity of the large cells in that their nuclei are almost spherical and have very loose heterochromatin as is typical of neuronal nuclei [90]. However, the failure of WGA, which has been established as a marker of neurons residing in the brain of *P*. *argus* [90], to label the large cells strongly indicates that they do not represent CNS neurons. This leaves the possibility that they represent a specialized population of sensory neurons (which also fail to label with WGA: M. Schmidt, unpublished). A sensory function of these cells would well be in line with the expression of the chemoreceptor protein IR25a –but what this function may be is enigmatic and would have to be addressed by recording the activity of the large cells with electrophysiological or imaging methods. Sensory neurons with somata located in the CNS have been observed in arthropods, albeit very rarely [125, 126]. In being located within a nerve root, the large cells to some extent resemble neurons located in nerve roots of the thoracic ganglia of *H*. *americanus* [127, 128]. These cells are octopaminergic, have processes arborizing in the connective tissue sheath surrounding the nerves, and serve a neuromodulatory function. Given that in adult spiny lobsters new ORNs are continually being born and innervating the olfactory lobe [129], it is tempting to speculate that these IR25a positive cells in the axon sorting zone might act in guiding or sorting of the axons of the new ORNs as they extend their processes into targets in the OL. This intriguing hypothesis deserved further examination.

In fact, our PCR identified iGluRs and IRs, including IR25a, not only in sensory organs and brain but also in the green gland of *P*. *argus*. The function of IRs in this excretory organ also needs further study, but this, together with our finding of IRs in the brain, suggests multiple roles for IRs.

### Crustacean GRs

We identified a partial sequence of a GR in the LF of *P*. *argus* (Fig 9). GRs having expanded families and demonstrated or putative chemosensory function have now been identified in insects, some crustaceans (most notably *Daphnia*, with its 58 GRs), chelicerates (ticks), and myriapods (centipedes) [13, 14, 31, 36, 38, 51, 55]. The GRs and their non-arthropod homologues, the GRLs, have ancient origin, and they appear to have been lost in some groups and expanded in others [13, 14, 31]. GRs are not just contact chemoreceptor proteins in *D*. *melanogaster* but are involved in other sensory functions such as promoting the detection of CO_2_, sensing fructose in hemolymph, detecting light, and sensing warm temperatures. GR28B(D), together with TRPA1, mediates thermotaxis [130–136]. GRLs in ancestral protostomes appear to have roles in development [51], and their selective expansion in some clades appears to be related to chemosensory functions [13, 14, 31]. GRs have been found in some crustaceans besides *Daphnia*, but not in high numbers–one in the barnacle *Amphibalanus amphitrite*, two in the copepod *Tigriopus californicus*, and six in the copepod *Eurytemora affinis* species [31]–and all are of unknown function. Our identification of only one GR in *P*. *argus* suggests that while present in this species, it is unlikely to play a role in chemoreception, or at least in the discrimination of diverse chemical stimuli. Determining the cellular expression patterns of this GR will help in elucidating possible functions. In addition, transcriptomic analysis of additional crustaceans and non-insect arthropods should help us understand the evolution and function of the GRL/GR gene family in this clade [9].

### Crustacean TRP channels

Homologues of all subfamilies of TRP channels [56, 57] were found in our *P*. *argus* transcriptome (Fig 10). Here, we discuss only the four subfamilies of TRP channels that have known chemosensory functions in other species. First is the TRPA homologues, including those related to TRPA1, painless, TRPA5, pyrexia, and waterwitch of insects, found in LF, dactyl, and brain. The expansion of the TRPA subfamily in *P*. *argus* with nine different types is similar to insects. Second is the TRPV homologues, including those related to OSM-9 of *C*. *elegans*, and Nanchung and Inactive of *D*. *melanogaster*. Third is the TRPC homologues in LF, dactyl, and brain. Fourth is the TRPM homologues, including those related to insect TRPM channels and mammalian TRPM channels, in LF, dactyl, and brain.

Although the types and numbers of *P*. *argus* TRP channels are similar to insects, there are a few differences in crustaceans. The crustaceans *P*. *argus*, *H*. *americanus*, and *C*. *borealis* have 2–3 types of TRPM channels, whereas insects have only one (Fig 10; [63–65, 85]). While *Daphnia* also has two TRPM channels, they are both cluster with the insect TRPM channels (Fig 10; [63]). One TRPM channel expressed in the LF, dactyl, and brain of *P*. *argus* is homologous to TRPM channel of insects, while the other TRPM homologue, PargTRPMm, is highly expressed in the LF and dactyl and is more closely related to mammalian TRPM channels. It is possible that with the inclusion of other decapod crustaceans in a phylogenetic analysis, PargTRPMm may turn out to be a crustacean specific TRPM channel and not a true homologue of mammalian TRPM channels.

The cellular expression patterns of TRP channels in *P*. *argus* were not explored, so their role in chemoreception remains speculative. We note that a sodium/calcium gated cation channel in ORNs of *P*. *argus* has certain physiological and pharmacological properties that resemble a TRPC channel [137–139]. However, the sequence of this channel has not been reported, and the only sequences of crustacean TRP channels that we have found in public databases are for TRPA, TRPM, and TRPV channels from transcriptomes of the central nervous system of *C*. *borealis* and *H*. *americanus* [64, 65] and all the TRP channels from the *D*. *pulex* genome [63].

Many temperature sensitive, or thermo, TRPs, which include TRP channels TRPV1, TRPV2, TRPV3, TRPV4, and TRPM8 in mammals and TRPA1 across animals, are also activated by irritants or noxious chemicals such as reactive oxygen or nitrogen species (ROS or RNS) [140, 141], electrophiles such as allyl isothiocyanate (AITC), capsaicin, menthol, and camphor. The diversity of the structures of these thermo TRP activating molecules suggests various activation mechanisms that are not restricted to the receptor-ligand model that is commonly observed in most chemoreceptor proteins. TRPA1 channels can be activated following covalent modification of their cysteine residues by membrane permeable molecules such as AITC, nitric oxide, RNS, or H_2_O_2_ [140–144]. In fact, Arenas et al. (2017) [141] discovered that planarian and human TRPA1 channels can rescue *D*. *melanogaster* TRPA1 function in mutants despite planarians, flies, and humans being separated by millions of years of evolution, due to the shared commonality of being activated by ROS and H_2_O_2_. Although TRPC channels are not known to be thermo TRPs, they have been implicated in chemical sensing, and several TRPC channels are known to be sensitive to NO and H_2_O_2_ [140]. Previous work has shown that H_2_O_2_ and other products found in opaline glands of the sea hare, *Aplysia californica*, are aversive and distasteful to *P*. *argus* [145]. The receptor proteins mediating this aversion response are unknown, but RNS/ROS and H_2_O_2_ sensitive receptors such as TRPA1 and TRPC channels, both of which are in our Parg transcriptomes, are candidates.

### Other chemoreceptor proteins in crustaceans?

We searched for homologues of other chemoreceptor proteins in our transcriptome. We did not find ORs in *P*. *argus*, which is consistent with past failures to identify ORs in non-insect arthropods, including crustaceans. Our results support the conclusion that ORs evolved after the origin of insects [146, 147]. We also searched for an expanded set of nicotinic acetylcholine receptors (nAChRs), such as is present in the *Octopus* genome and which speculatively might be a set of chemoreceptor proteins [148]. While we found several nAChR homologues, we did not find evidence of an expanded family. Another class of chemoreceptor proteins, vertebrate-like ORs, are 7TM GPCRs. While there are several rhodopsin-like GPCRs in the *P*. *argus* transcriptome, our initial InterProScan and BLAST searches did not reveal any chemosensory GPCRs. Perhaps the inclusion of other decapods in phylogenetic analyses may resolve some of these 7TM GPCRs to be chemosensory.

## Conclusions

The Caribbean spiny lobster, *P*. *argus*, has at least 108 IRs in two of its major chemosensory organs, the LF and dactyls of legs. Of these 108 IRs, four are co-receptor IRs and one is a conserved IR that are expressed in both LF and leg dactyl. The other 95 are divergent IRs, and most (51) are expressed only in the LF. Supporting the role of these IRs in chemoreception is the fact that the co-receptor IR25a is expressed in chemosensory cells in these organs–the ORNs of the LF and CRNs in the dactyls and LF–but they are not expressed in MRNs in either organ. It is interesting, though, that the co-receptor IRs and conserved IRs are expressed in other tissues including brain, which may be related to their demonstrated role in insects in non-chemosensory functions. Besides IRs, we found one GR and 18 TRP channels in *P*. *argus*, though any function in chemoreception in *P*. *argus* is unknown at this time.

Many questions remain unanswered. The much higher number of divergent IRs in the LF compared to dactyls is correlated with the unique role of LF in detecting waterborne conspecific cues including social, alarm, and sex cues, in addition to their detecting feeding cues also sensed by the dactyls. However, the chemical specificity of individual IRs needs to be determined to identify their broader functional roles. Related to this is the need to determine the expression patterns of IRs in individual cells. For example, how many IRs are co-expressed in individual ORNs or CRNs, and what is the diversity of expression patterns in these cells? Each aesthetasc of *P*. *argus* is innervated by ca. 300 ORNs with a diversity of chemical specificities, such that the aesthetasc is considered to be a functional unit of olfaction [86, 149, 150]. Correlating IR expression pattern with chemical specificity for individual ORNs will be important in determining rules of olfactory coding in the periphery.

How many different types of chemoreceptor proteins exist in a crustacean? For *P*. *argu*s, we have sampled only two sensory organs, and the conservative answer is nearly 130, including IRs, GRs, and TRP channels. Given that the IR populations in *P*. *argus* are largely non-overlapping in LF and dactyls, it might be expected that when other chemosensory organs are analyzed, the total number of chemoreceptor proteins in *P*. *argus* will be much higher. How does this compare with other crustaceans? Analysis of the genome of *Daphnia* reveals at least 143 chemoreceptor proteins: 58 GRs and 85 IRs [13, 25, 27, 55]. Two species of copepods have been reported to have 8 IRs and 2–6 GRs [31]. Transcriptomes of the antennule from two species of hermit crabs have yielded up to 29 IRs and no ORs or GRs per species [42, 43]. A better understanding of the olfactory logic in crustaceans, including differences associated with phylogeny, sex, or development, will benefit from an examination of more crustacean species.

Finally, important questions remain unanswered regarding how the chemoreceptor proteins are represented in the spiny lobster’s central nervous system through the central projections of ORNs. The antennule’s 300,000 ORNs with their ca. 96 divergent IRs project into ca. 1200 glomeruli in the OL of the brain. This suggests the possibility of an olfactory wiring logic in decapod crustaceans that is significantly different than in insects or mammals, in which the ratio of the number of types of receptor molecules to glomeruli is 1:1 [151–153].

## Supporting information

S1 Table Primers. (XLSX)

S2 Table RSEM gene counts matrix of variant IRs in Parg transcriptome. (XLSX)

S3 Table RSEM gene counts matrix of TRP channels in Parg transcriptome. (XLSX)

S1 Text Trinity assembly statistics for Parg transcriptome. (TXT)

S2 Text BUSCO output before and after cd-hit on Parg transcriptome. (TXT)

S3 Text Parg_PF00060. (FASTA)

S4 Text Parg_PF10613. (FASTA)

S5 Text Selected_IRs_Figs 2 and 3. (FASTA)

S6 Text MAFFTalnd_selected_IRs_Figs 2 and 3. (FASTA)

S7 Text Trimmed_MAFFTalnd_selected_IRs_Fig 2. (FASTA)

S8 Text ConsvIRs_Fig 4. (FASTA)

S9 Text MAFFTalnd_ConsvIRs_Fig 4. (FASTA)

S10 Text Trimmed_MAFFTalnd_ConsvIRs_Fig 4. (FASTA)

S11 Text ArthropodGRs_Fig 9. (TXT)

S12 Text MAFFTalnd_ArthropodGRs_Fig 9. (FASTA)

S13 Text TRPchannels_Fig 10. (FASTA)

S14 Text MAFFTalnd_TRPchannels_Fig 10. (FASTA)

S15 Text Trimmed_MAFFTalnd_TRPchannels_Fig 10. (FASTA)

S1 Fig Heatmap of abundance of IRs in the LF, dactyl, and brain based on non-normalized raw counts. A qualitative representation of raw counts from RSEM.gene.counts.matrix generated by RSEM perl script in Trinity. Plot was created with heatmap.2 function from R gplot package.
(PDF)

S2 Fig Heatmap of abundance of TRP channels in the LF, dactyl, and brain based on non-normalized raw counts. A qualitative representation of raw counts from RSEM.gene.counts.matrix generated by RSEM perl script in Trinity. Plot was created with heatmap.2 function from R gplot package.
(PDF)
