# Supplementary figures and images for: Chemoreceptor proteins in the Caribbean spiny lobster, Panulirus argus: Expression of Ionotropic Receptors, Gustatory Receptors, and TRP channels in two chemosensory organs and brain
Source: PLoS One. 2018 Sep 21;13(9):e0203935. doi: 10.1371/journal.pone.0203935 (PMC6150509; doi:10.1371/journal.pone.0203935)

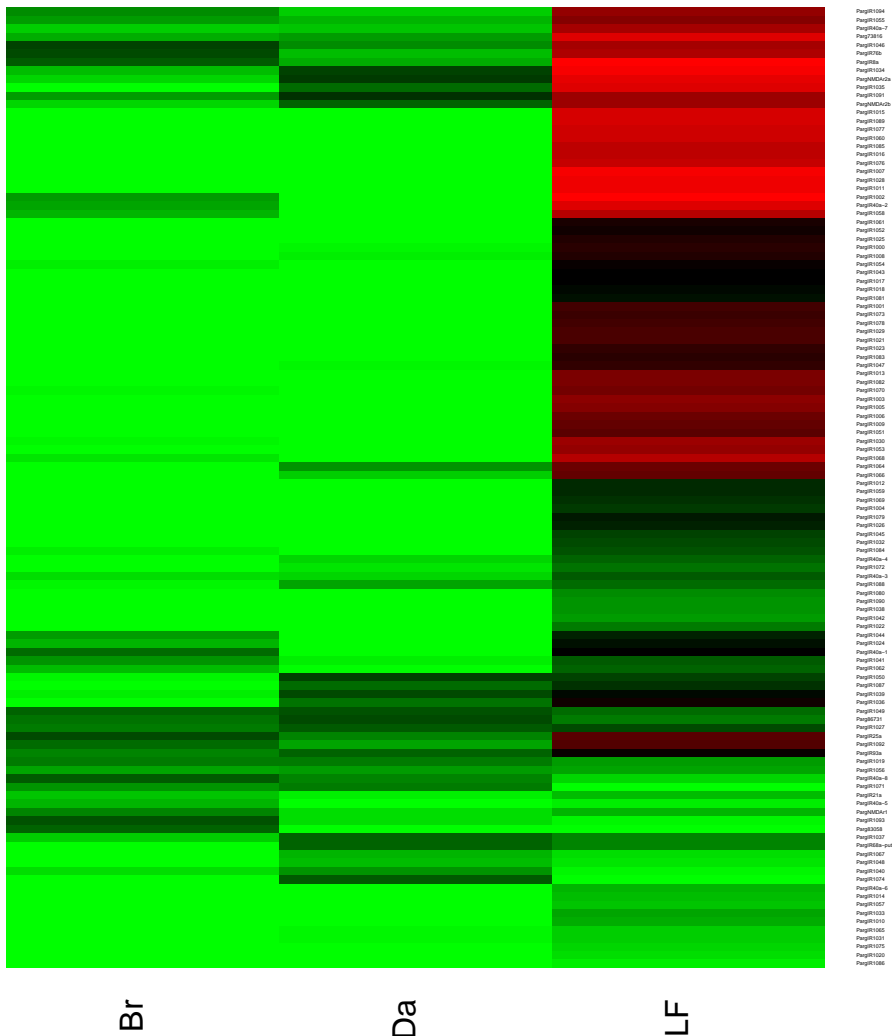

Supplement: S1 Fig — A qualitative representation of raw counts from RSEM.gene.counts.matrix generated by RSEM perl script in Trinity. Plot was created with heatmap.2 function from R gplot package. (PDF) [file pone.0203935.s019.pdf]

# Color Key

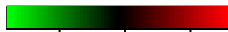

5

10

15

Value

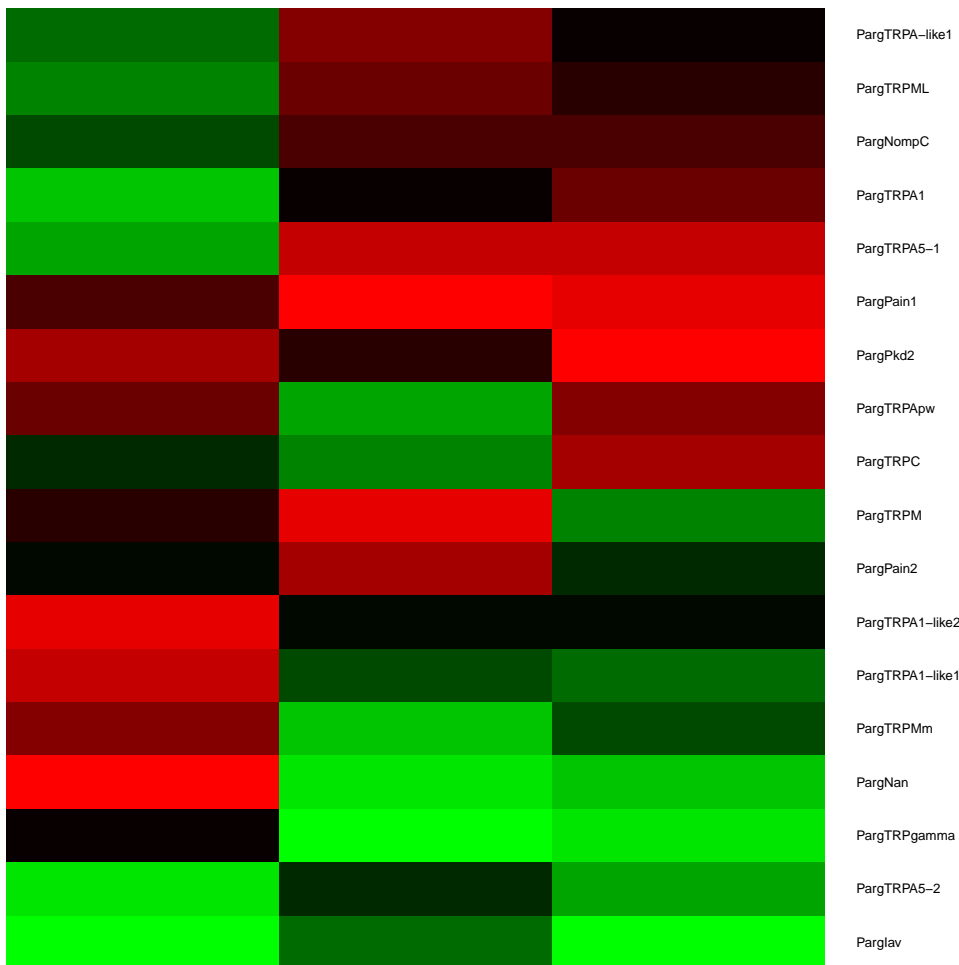

LF

Da

Br

Supplement: S2 Fig — A qualitative representation of raw counts from RSEM.gene.counts.matrix generated by RSEM perl script in Trinity. Plot was created with heatmap.2 function from R gplot package. (PDF) [file pone.0203935.s020.pdf]
